# Vision protection and robust axon regeneration in glaucoma models by membrane-associated Trk receptors

**DOI:** 10.1016/j.ymthe.2022.11.018

**Published:** 2022-12-05

**Authors:** Euido Nishijima, Sari Honda, Yuta Kitamura, Kazuhiko Namekata, Atsuko Kimura, Xiaoli Guo, Yuriko Azuchi, Chikako Harada, Akira Murakami, Akira Matsuda, Tadashi Nakano, Luis F. Parada, Takayuki Harada

**Affiliations:** 1 Visual Research Project, Tokyo Metropolitan Institute of Medical Science, Setagaya-ku, Tokyo 156-8506, Japan; 2 Department of Ophthalmology, The Jikei University School of Medicine, Minato-ku, Tokyo 105-8461, Japan; 3 Department of Ophthalmology, Juntendo University Graduate School of Medicine, Bunkyo-ku, Tokyo 113-8421, Japan; 4 Department of Ophthalmology and Visual Science, Chiba University Graduate School of Medicine, Chiba, Chiba 260-8670, Japan; 5 Brain Tumor Center and Cancer Biology & Genetics Program, Memorial Sloan Kettering Cancer Center, New York, NY 10065, USA

**Keywords:** TrkB, gene therapy, glaucoma, retinal ganglion cell, neuroprotection, dendrite, synapse, axon regeneration, optic nerve injury, optic tract transection

## Abstract

Activation of neurotrophic factor signaling is a promising therapy for neurodegeneration. However, the transient nature of ligand-dependent activation limits its effectiveness. In this study, we solved this problem by inventing a system that forces membrane localization of the intracellular domain of tropomyosin receptor kinase B (iTrkB), which results in constitutive activation without ligands. Our system overcomes the small size limitation of the genome packaging in adeno-associated virus (AAV) and allows high expression of the transgene. Using AAV-mediated gene therapy in the eyes, we demonstrate that iTrkB expression enhances neuroprotection in mouse models of glaucoma and stimulates robust axon regeneration after optic nerve injury. In addition, iTrkB expression in the retina was also effective in an optic tract transection model, in which the injury site is near the superior colliculus. Regenerating axons successfully formed pathways to their brain targets, resulting in partial recovery of visual behavior. Our system may also be applicable to other trophic factor signaling pathways and lead to a significant advance in the field of gene therapy for neurotrauma and neurodegenerative disorders, including glaucoma.

## Graphical abstract

**Figure:**
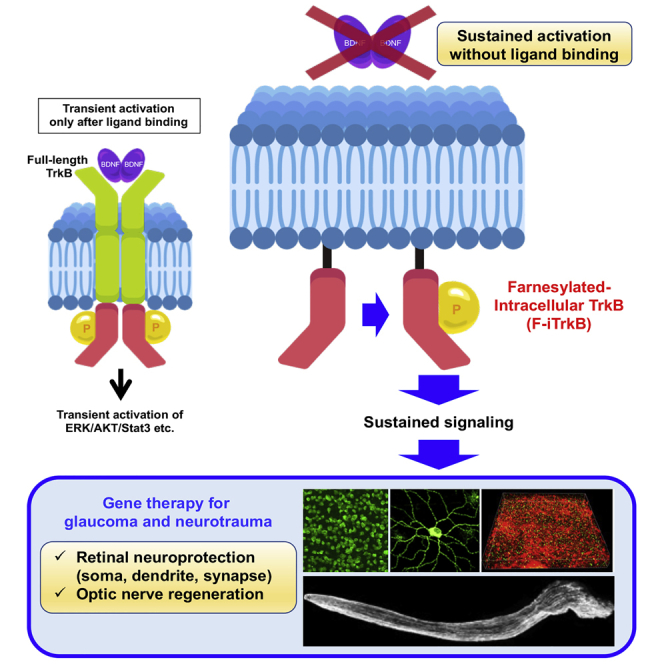

## Teaser

Harada and colleagues developed a system that forces membrane localization of the intracellular domain of TrkB (iTrkB), which results in constitutive activation without ligands. AAV-mediated delivery of iTrkB into the eyes induced neuroprotection and robust optic nerve regeneration in mouse models of glaucoma and optic nerve injury.

## Introduction

Neurodegenerative diseases are debilitating conditions characterized by cognitive and/or motor impairment due to progressive loss of neural function or neuron death. Among them, glaucoma is the leading cause of irreversible blindness due to optic nerve damage and the death of retinal ganglion cells (RGCs). The optic nerve is composed of RGC axons, which transmit visual information from the eyes to brain targets, such as the superior colliculus (SC) and lateral geniculate nucleus. Currently, the reduction of intraocular pressure (IOP) is the sole evidence-based therapy for glaucoma patients, but it is ineffective in a considerable proportion of glaucoma patients, especially those with normal tension glaucoma.^1^^,^^2^ Thus, other strategies for suppressing further degeneration of RGCs, such as neuroprotection, have been investigated. Gene therapy is potentially an effective therapeutic approach for neurodegenerative diseases, and tools for delivering genes into injured RGCs have been explored.^3^^,^^4^ Indeed, adeno-associated virus (AAV) delivery of some trophic factors, such as brain-derived neurotrophic factor (BDNF) or ciliary neurotrophic factor (CNTF), stimulates the protection and axon regeneration of RGCs in a mouse model of optic nerve injury.^5^^,^^6^^,^^7^ However, the concentration of such molecules that are exogenously applied is rapidly decreased by diffusion or metabolism, inhibiting sustained signal transduction. Furthermore, the number of receptors available at the cell surface may limit the strength of signal transduction by such trophic factors. Another problem is that the packaging capacity of AAV is limited to ∼4.7 kb; thus, some adjustment to the size of promoters and/or molecules will be required to express a large molecule at a high expression level. To overcome these problems, a new idea for effective and continuous stimulation of trophic factor signaling is required. Tropomyosin receptor kinase B (TrkB) is a high-affinity neurotrophin receptor for BDNF that activates several intracellular signaling pathways to promote cell growth and survival upon BDNF binding. Because the extracellular domain of TrkB possesses an autoinhibitory domain,^8^ we speculate that the membrane-bound intracellular domain of TrkB could induce downstream signaling without BDNF.

Protein lipidation, which includes farnesylation, myristoylation, palmitoylation, and geranylgeranylation, is a method for localizing proteins at the cell membrane by covalent attachment of a lipophilic group. Of these, farnesylation is a form of posttranslational prenylation modification that involves the attachment of a farnesyl group to the C-terminal cysteine residue of the target protein, facilitating membrane association and protein-protein interactions.^9^^,^^10^ In this study, we generated a constitutive active form of TrkB by farnesylation of the intracellular domain of TrkB (F-iTrkB). The relatively small size of F-iTrkB allowed for the use of the most powerful form of a CAG promoter in an AAV vector, which generated a high expression of F-iTrkB. Intraocular injection of AAV-F-iTrkB promoted RGC protection and robust axon regeneration without exogenous BDNF application. Our results indicate that artificial lipidation of the intracellular domains of trophic factor receptors triggers powerful signal transduction, which may be effective as a gene therapy tool for neurodegenerative diseases.

## Results

### Membrane-anchored intracellular TrkB elicits ligand-independent signaling

**Figure 1:**
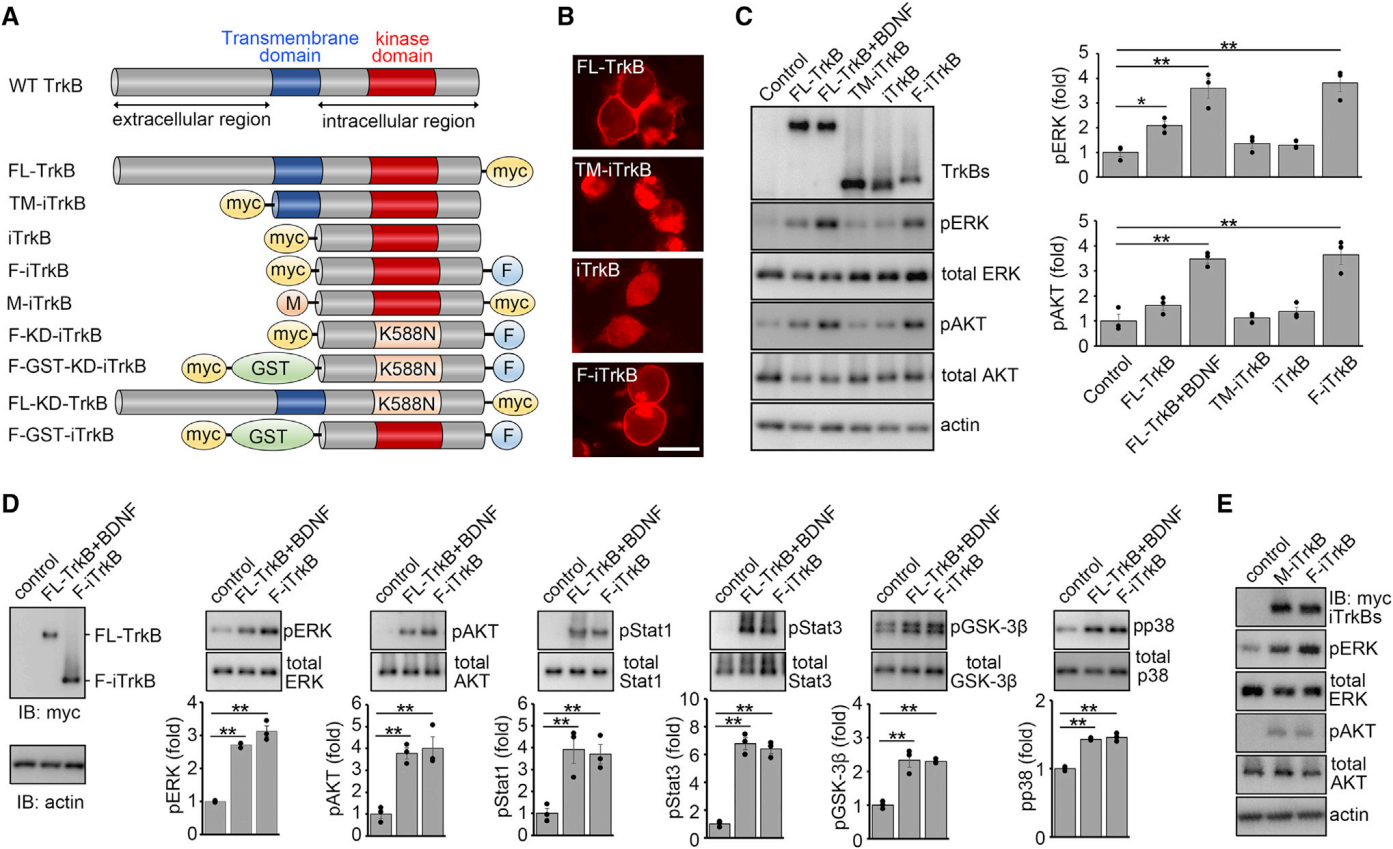
Farnesylated intracellular domain of TrkB activates downstream signaling without ligands (A) Schematic diagram of TrkB constructs used in this study. TM, transmembrane domain; myc, myc-tag; F, farnesylation signal; GST, glutathione S-transferase; K588N, substitution of lysine with asparagine at position 588, to generate a kinase-dead form. (B) Cellular localization of TrkB mutants. Immunostaining of myc-tagged TrkB mutants in Neuro2A cells transfected with the indicated plasmids. Full-length (FL) TrkB and farnesylated intracellular domain of TrkB (F-iTrkB) were localized at the peripheral region. Scale bar, 25 μm. (C) Activation of ERK and AKT by TrkB mutants. Immunoblot analysis of ERK and AKT phosphorylation in Cos-7 cells transfected with the indicated plasmids. Representative immunoblot images (left) and quantification of the relative levels of phosphorylated ERK (pERK) and AKT (pAKT) (right). The one-way ANOVA with Tukey-Kramer *post hoc* test was used. n = 3 per experimental condition. ∗p < 0.05, ∗∗p < 0.01. (D) Comparison of ligand-stimulated FL-TrkB and F-iTrkB in activation of downstream signaling. Immunoblot analysis of several signal proteins in Cos-7 cells transfected with FL-TrkB with BDNF stimulation for 20 min (FL-TrkB + BDNF) or F-iTrkB. TrkB and actin expression is shown (left). pERK, pAKT, pStat1, pStat3, pGSK-3β, and pp38 were detected in both groups. Representative immunoblot images are shown (top), and the relative levels of phosphorylated proteins were quantified (bottom). The one-way ANOVA with Tukey-Kramer *post hoc* test was used. n = 3 per experimental condition. ∗∗p < 0.01. (E) The effect of myristoylation and farnesylation on the activity of iTrkB. Cos-7 cells were transfected with myristoylated iTrkB (M-iTrkB) or F-iTrkB. pERK and pAKT were detected from both mutants.

We first explored developing constitutively active TrkB. When BDNF binds to TrkB, TrkB dimerizes, and each molecule transphosphorylates the other, activating downstream signaling pathways, such as Ras-ERK and PI3K-AKT. It was previously reported that a TrkB mutant lacking immunoglobulin domains activated ERK in HEK293 cells without BDNF, but the activity of the TrkB mutant was very low compared with wild-type (WT) TrkB stimulated by BDNF.^8^ We prepared several TrkB mutants to develop a constitutively active TrkB molecule that induces powerful signal activation (Figure 1A). Analysis of cellular localization revealed that the full-length TrkB (FL-TrkB) was detected at the cell periphery in Neuro2A cells, whereas the intracellular domain of TrkB (iTrkB) and iTrkB with the transmembrane domain (TM-iTrkB) were expressed diffusely in the cytoplasm (Figure 1B). These observations prompted us to design a TrkB mutant that promotes intracellular membrane anchoring by attaching the farnesylation signal sequence CAAX (F-iTrkB) (Figure 1A). We discovered that F-iTrkB, like FL-TrkB, was localized at the membrane of Neuro2A cells (Figure 1B). We examined the signal transduction activities of these TrkB mutants using immunoblot analysis (Figure 1C). TM-iTrkB and iTrkB failed to activate ERK and AKT, whereas F-iTrkB strongly induced phosphorylation of ERK (pERK) and AKT (pAKT) compared with FL-TrkB alone. In addition, the levels of pERK and pAKT induced by F-iTrkB were similar to those induced by FL-TrkB stimulated with exogenous BDNF (FL-TrkB + BDNF). Furthermore, F-iTrkB expression increased the phosphorylation of other signaling molecules, including Stat1, Stat3, GSK-3β, and p38, as observed with FL-TrkB + BDNF (Figure 1D). We also discovered that myristoylated iTrkB (M-iTrkB) activates ERK and AKT at levels similar to those by F-iTrkB (Figure 1E).

**Figure 2:**
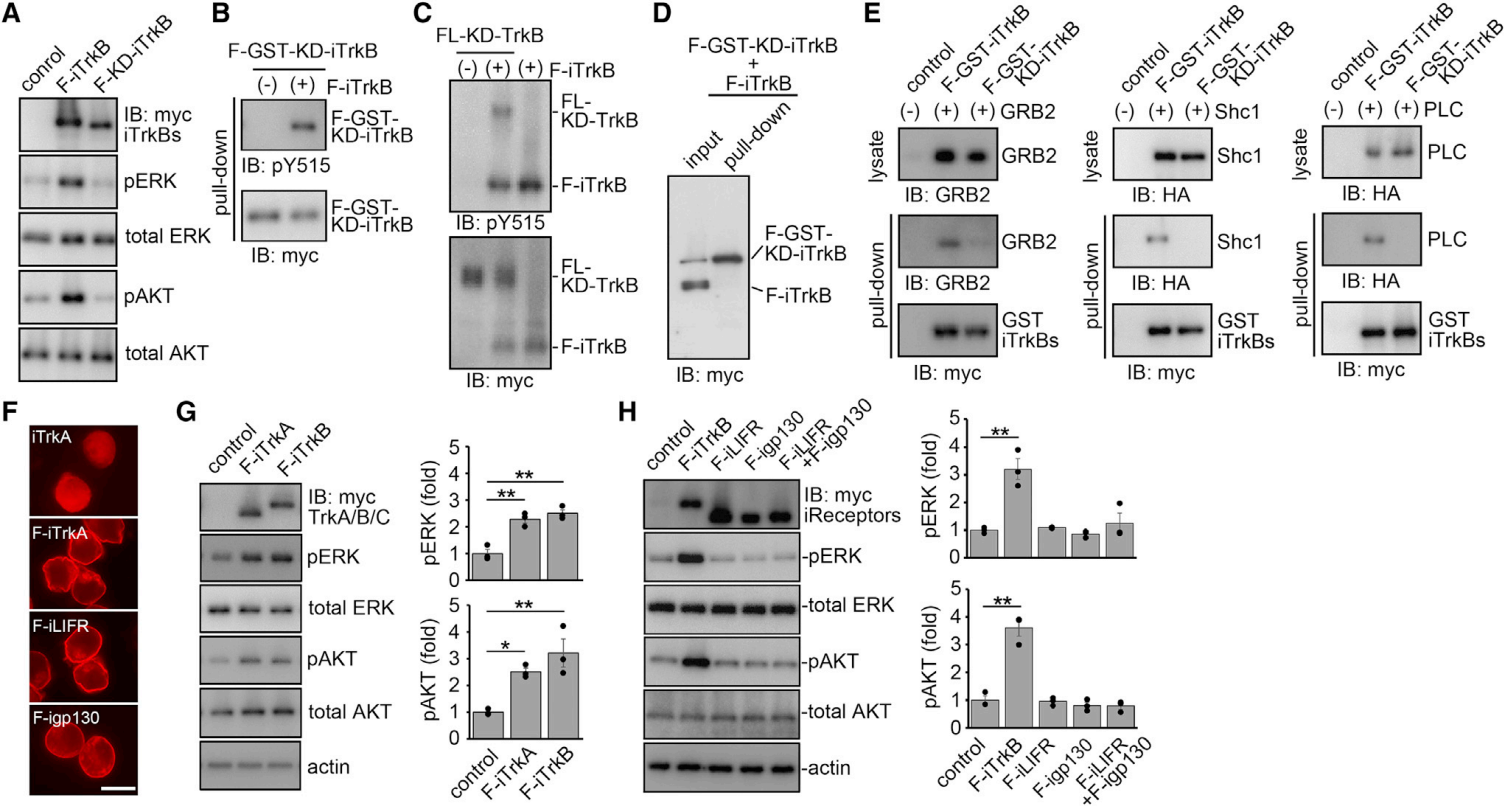
Characterization of F-iTrkB and farnesylated receptors of other trophic factors (A) The effect of kinase activity of F-iTrkB on signal activation. Immunoblot analysis of pERK and pAKT in Cos-7 cells transfected with F-iTrkB or F-KD-iTrkB. Representative images are shown. (B) Transphosphorylation of F-iTrkB. Cos-7 cells were cotransfected with a kinase-dead (KD) form of GST-tagged F-iTrkB (F-GST-KD-iTrkB) and F-iTrkB, followed by a GST pull-down assay. The pull-down sample was subjected to immunoblot analysis using an anti-phospho-TrkB (pY515) antibody. (C) F-iTrkB-mediated phosphorylation of FL-TrkB. Cos-7 cells were transfected with a KD form of the FL-TrkB (FL-KD-TrkB), alone or cotransfected with F-iTrkB. Total cell lysates were analyzed by immunoblotting. (D) No detection of F-iTrkB dimers. Cos-7 cells were cotransfected with a KD form of GST-tagged F-iTrkB (F-GST-KD-iTrkB) and F-iTrkB, followed by a GST pull-down assay. The pull-down sample was subjected to immunoblot analysis. (E) Interaction of F-iTrkB with GRB2, Shc, and PLC. Cos-7 cells were cotransfected with F-GST-iTrkB and GRB2 (left), HA-tagged Shc1 (middle), or HA-tagged PLC (right), followed by a GST pull-down assay. The pull-down sample was subjected to immunoblot analysis. (F) Cellular localization of iTrkA, F-iTrkA, F-igp130, and F-iLIFR. Immunostaining of myc-tagged proteins in Neuro2A cells transfected with the indicated plasmids. Farnesylated proteins were localized at the peripheral region. Scale bar, 25 μm. (G) F-iTrkA-mediated activation of ERK and AKT. Immunoblot analysis of ERK and AKT phosphorylation in Cos-7 cells transfected with F-iTrkA or F-iTrkB. Representative images (left) and quantification of the relative levels of pERK and pAKT (right). The one-way ANOVA with Tukey-Kramer *post hoc* test was used. n = 3 per experimental condition. ∗p < 0.05, ∗∗p < 0.01. (H) Absence of signal activation by farnesylated intracellular domain of cytokine receptors. Immunoblot analysis of ERK and AKT phosphorylation in Cos-7 cells transfected with F-iLIFR and F-igp130 and a mixture of the two plasmids. Representative images (left) and quantification of the relative levels of pERK and pAKT (right). The one-way ANOVA with Tukey-Kramer *post hoc* test was used. n = 3 per experimental condition. ∗∗p < 0.01.

Because F-iTrkB demonstrated powerful activation of multiple downstream signaling without BDNF, we further elucidated its properties. First, we investigated whether the kinase activity of TrkB is essential for F-iTrkB-mediated ERK and AKT activation. Immunoblot analysis revealed that a kinase-dead mutant (F-KD-iTrkB) failed to phosphorylate ERK and AKT, indicating that the kinase activity is essential for F-iTrkB-mediated signal transduction (Figure 2A). We constructed a GST-fused kinase-dead form of F-iTrkB (F-GST-KD-iTrkB) to investigate whether transphosphorylation between F-iTrkB occurs. We discovered that F-GST-KD-iTrkB was phosphorylated at Tyr515 in the presence of F-iTrkB, but not in its absence (Figure 2B), demonstrating that F-iTrkB transphosphorylated F-GST-KD-iTrkB. In addition, a full-length kinase-dead TrkB (FL-KD-TrkB) was phosphorylated at Tyr515 by F-iTrkB, suggesting that F-iTrkB phosphorylates endogenous WT TrkB (Figure 2C). These results suggest that F-iTrkB transphosphorylates both endogenous WT TrkB and exogenous F-iTrkB at Tyr515. Because BDNF induces TrkB dimerization for transphosphorylation, we investigated whether F-iTrkB forms a dimer using Cos7 cells. A pull-down assay revealed that F-iTrkB does not bind to F-GST-KD-iTrkB (Figure 2D). These results indicate that F-iTrkB does not form a dimer, and transphosphorylation of F-iTrkB at Tyr515 was induced by a transient interaction. We also examined whether phosphorylated F-iTrkB is associated with GRB2, Shc, and PLC, like WT TrkB. Immunoblot analysis after a pull-down assay revealed that F-GST-iTrkB was phosphorylated at Tyr515 and bound to GRB2, Shc, and PLC, but not F-GST-KD-iTrkB (Figure 2E). These results indicate that F-iTrkB robustly stimulates downstream signaling without BDNF through the conventional BDNF-TrkB pathway without forming a stable dimer.

Since TrkA is also involved in the survival of neurons, such as RGCs,^11^ we examined the effect of F-iTrkA. F-iTrkA transfection in Neuro2A cells resulted in membrane localization, but not iTrkA (Figure 2F), and F-iTrkA activated downstream signaling like F-iTrkB (Figure 2G). Furthermore, we examined cytokine receptors, including gp130 and LIFR. To activate cytokine receptor signaling, including CNTF signaling, gp130 and LIFR form a complex. Therefore, we generated F-igp130 and F-iLIFR plasmid constructs. We discovered that transfection of F-igp130 or F-iLIFR or the combination of both (F-igp130 + F-iLIFR) failed to activate ERK and AKT, which function downstream of CNTF signaling (Figure 2H). Collectively, these data demonstrate that membrane localization of the intracellular region of TrkA and TrkB activates their downstream signaling pathways in the absence of ligands.

### Overexpression of F-iTrkB alters gene expression in retinal ganglion cells

**Figure 3:**
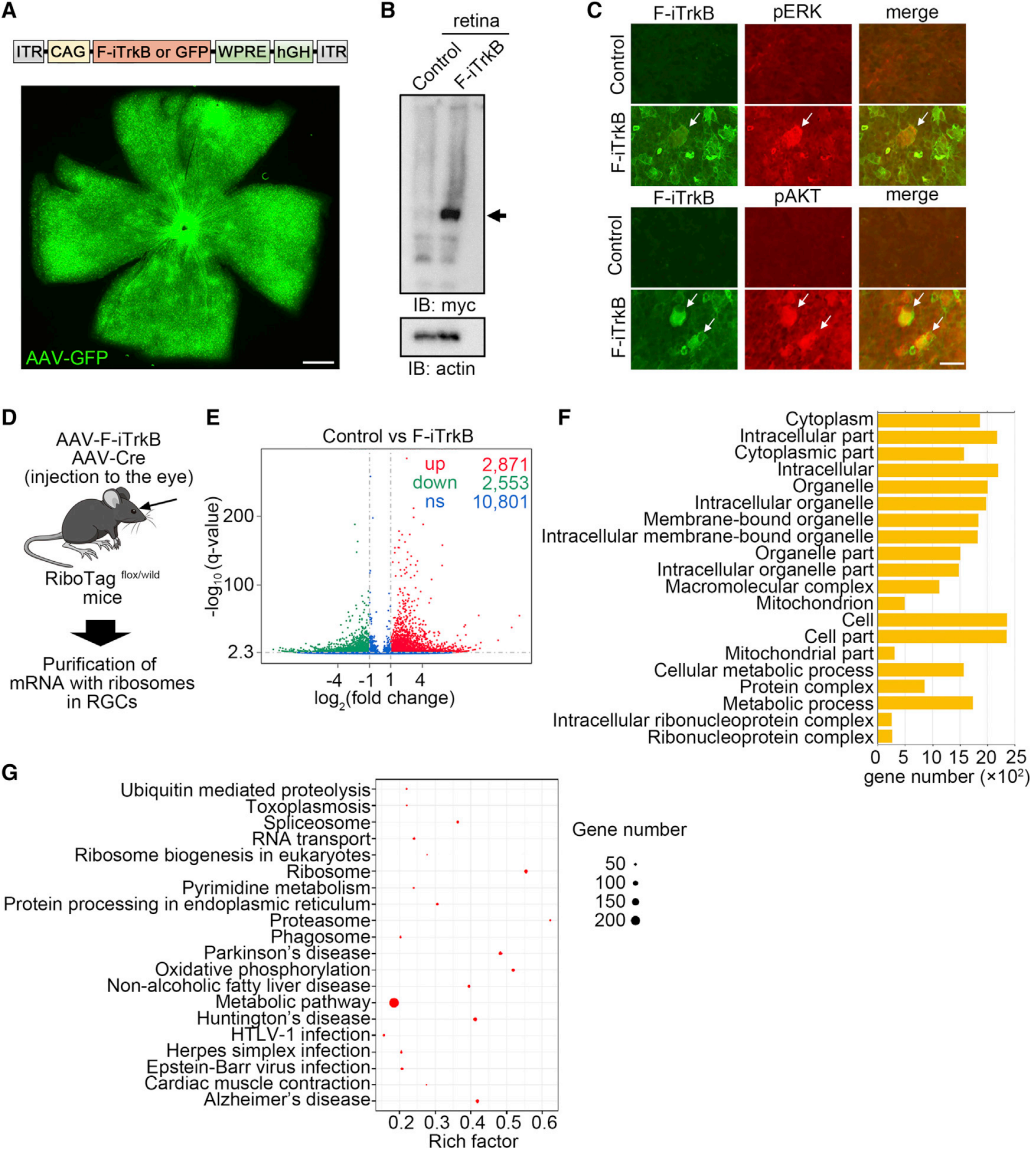
Intravitreal injection of AAV-F-iTrkB alters gene expression in RGCs (A) Schematic diagram of the plasmid construction for AAV (top). AAV transduction of cells in the mouse retina was performed. AAV-GFP was intravitreally injected into WT mice. Two weeks after injection, GFP expression was detected in the flat-mounted retina (bottom). Scale bar, 300 μm. (B) Expression of F-iTrkB in the mouse retina. AAV-F-iTrkB was intravitreally injected into WT mice. Two weeks after injection, expression of myc-tagged F-iTrkB (arrow) was detected in retinal homogenates by immunoblotting. (C) F-iTrkB-mediated ERK and AKT activation in RGCs (arrows). Double-immunostaining of retinal flat mounts using anti-myc (for F-iTrkB, green) and anti-pERK (top, red) or anti-pAKT (bottom, red) antibodies is shown. (C) F-iTrkB-mediated ERK and AKT activation in RGCs. Double-immunostaining of retinal flat mounts using anti-myc (for F-iTrkB, green) and anti-pERK (top, red) or anti-pAKT (bottom, red) antibodies is shown. Scale bar, 25 μm. (D) Schematic diagram of the protocol for purification of RGC-specific RNA. (E) Volcano plot of gene expression in RGCs infected with AAV-F-iTrkB. The red dots represent significantly upregulated genes, the green dots represent significantly downregulated genes (|log2(fold change)| > 1 and q < 0.005), and the blue dots represent gene expression with no significant difference between the treatment group (AAV-F-iTrkB) and the control group (AAV-Control). (F) Gene Ontology (GO) analysis of differentially expressed genes between AAV-Control and AAV-F-iTrkB-treated RGCs. The most enriched 20 GO terms among the upregulated genes are shown. (G) Kyoto Encyclopedia of Genes and Genomes (KEGG) enrichment analysis of differentially expressed genes between AAV-Control and AAV-F-iTrkB-treated RGCs. The top 20 most significantly enriched KEGG pathways are shown.

Death of or damage to RGCs is observed in glaucoma, the second leading cause of blindness globally. BDNF-TrkB signaling plays key physiological roles in the protection of neurons, including RGCs, and decreased expression levels of BDNF and TrkB are observed in the optic nerve head tissues from glaucoma patients.^12^^,^^13^^,^^14^ Indeed, we discovered accelerated glaucomatous RGC and optic nerve degeneration in aged mice that lack TrkB in neurons (TrkB^c-kit^ knockout [KO] mice) (Figure S1).^12^ These findings prompted us to investigate the neuroprotective effects of F-iTrkB on RGCs *in vivo*. For this purpose, we prepared an AAV serotype 2-based vector to express F-iTrkB (AAV-F-iTrkB) or GFP (AAV-GFP) as the control (Figure 3A). Two weeks after intraocular injection of AAV-GFP into the WT mice, we detected numerous GFP-positive cells in the retina (Figure 3A). Following the injection of AAV-F-iTrkB into the eye, immunoblot analysis revealed that F-iTrkB expression was detected in the retinal homogenate (Figure 3B). We performed immunohistological analysis to examine the ability of F-iTrkB to phosphorylate ERK and AKT *in vivo*. Two weeks after intravitreal injection of AAV-F-iTrkB, upregulation of pERK and pAKT was observed in cells expressing myc-tagged F-iTrkB (Figure 3C), indicating that F-iTrkB can induce signal transduction *in vivo*.

Next, we investigated the effects of F-iTrkB on gene expressions in RGCs. For this, we isolated mRNAs from RGCs using RiboTag mice (Figure 3D).^15^ In RiboTag mice, we intravitreally injected AAV-Cre, in combination with AAV-F-iTrkB or AAV-GFP for control. The hemagglutinin (HA)-tagged ribosome was purified by immunoprecipitation using an anti-HA antibody, and subsequently, the ribosome-bound RNA was purified. RNA-sequence analysis was performed using purified RNA, revealing that the expression of numerous genes was altered by F-iTrkB (Figure 3E). The number of upregulated genes was 2,871, while the number of downregulated genes was 2,553. Among the upregulated genes, the most enriched 20 Gene Ontology (GO) terms (Figure 3F) and the top 20 most significantly enriched Kyoto Encyclopedia of Genes and Genomes (KEGG) pathways are shown (Figure 3G). The data highlighted pathways associated with metabolic processes, such as mitochondrion, mitochondrial part, cellular metabolic process, and metabolic process, in GO analysis, and oxidative phosphorylation and metabolic pathways in KEGG analysis, implying that F-iTrkB may affect energy homeostasis in RGCs. Furthermore, gene categories associated with protein degradation and neurodegenerative diseases, such as ubiquitin-mediated proteolysis, proteasome, phagosome, Huntington’s disease, and Alzheimer’s disease, were identified in the KEGG analysis. These results suggest that F-iTrkB expression may also affect protein degradation and/or neurodegeneration.

### F-iTrkB protects RGCs in mouse models of glaucoma

**Figure 4:**
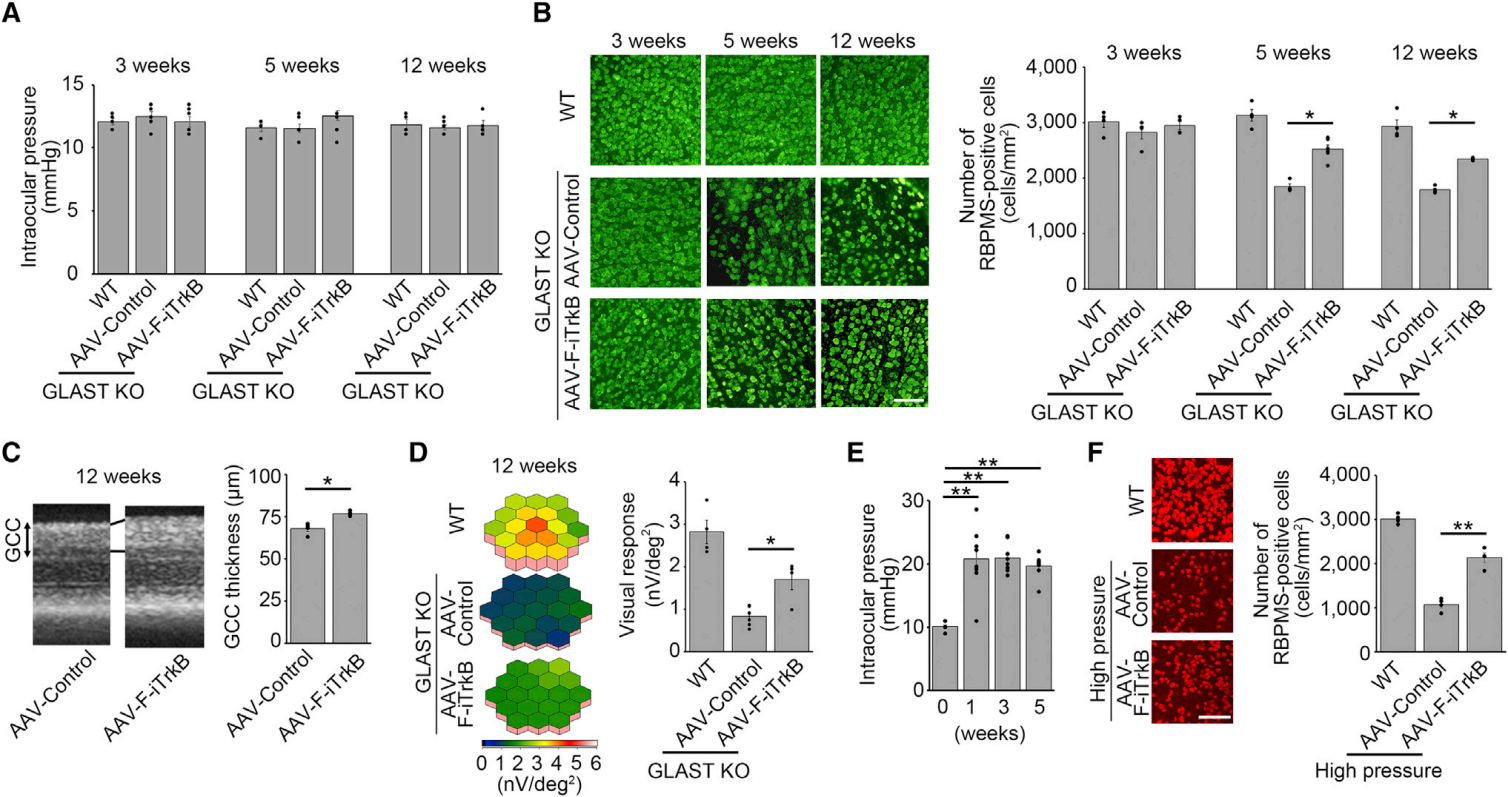
AAV-F-iTrkB prevents RGC degeneration in experimental models of glaucoma (A) Intraocular pressure (IOP) of WT or GLAST KO mice at 3, 5, and 12 weeks of age. AAV-F-iTrkB was intravitreally injected into GLAST KO mice at 10 days of age. n = 4–6 mice per group. (B) AAV-F-iTrkB-mediated RGC protection in GLAST KO mice. RGCs were detected by immunostaining of RBPMS. AAV-Control or AAV-F-iTrkB was intravitreally injected into GLAST KO mice at 10 days of age. Representative images (left) and quantification of RGCs (right) are shown. The one-way ANOVA with Tukey-Kramer *post hoc* test was used. n = 4–6 mice per group. ∗p < 0.05. Scale bar, 100 μm. (C) Optical coherence tomography (OCT) of GLAST KO mouse retinas treated with AAV-Control or AAV-F-iTrkB. Cross-sectional images of the retinas at 12 weeks of age (left) and quantification of the ganglion cell complex (GCC) thickness (right) are shown. Two-tailed unpaired Student’s t test was used. n = 4–6 mice per group. ∗p < 0.05. (D) Multifocal electroretinography (mfERG) of WT or GLAST KO mice treated with AAV-Control or AAV-F-iTrkB. Retinal responses of second-order kernel at 12 weeks of age are presented with 3D plot images (left) and quantitative analyses of the retinal response amplitude (right). The one-way ANOVA with Tukey-Kramer *post hoc* test was used. n = 4–6 mice per group. ∗p < 0.05. (E) IOP in WT mice with silicone oil-induced ocular hypertension. IOP is elevated by 1 week after the injection of silicone oil into the anterior chamber of the mouse eye. The one-way ANOVA with Tukey-Kramer *post hoc* test was used. n = 8 mice per group. ∗∗p < 0.01. (F) AAV-F-iTrkB-mediated RGC protection in mice with high IOP. RGCs were detected by immunostaining of RBPMS in WT mice at 4 weeks after silicone oil injection. Representative images (left) and quantification of RGCs (right) are shown. The one-way ANOVA with Tukey-Kramer *post hoc* test was used. n = 4 mice per group. ∗∗p < 0.01. Scale bar, 100 μm.

To investigate the neuroprotective effects of increased TrkB signaling on RGCs, we first employed a mouse model of normal tension glaucoma, glutamate/aspartate transporter (GLAST) KO mice. In GLAST KO mice, RGC degeneration occurs between 3 and 5 weeks of age, while maintaining normal IOP.^16^^,^^17^ The relatively fast disease time course is an advantage when evaluating novel therapies. IOP was comparable in GLAST KO and WT mice, and intraocular injection of AAV-F-iTrkB at postnatal day 10 did not affect IOP (Figure 4A). We then performed immunostaining of retinal flat mounts using an anti-RBPMS antibody (as a pan-RGC marker) and counted the number of RBPMS-positive cells, namely, RGCs.^18^ AAV-F-iTrkB treatment significantly enhanced the survival of RGCs in 5- and 12-week-old GLAST KO mice (Figure 4B). When we examined retinal morphology *in vivo* using optical coherence tomography (OCT), we discovered that the ganglion cell complex (GCC), which contains the RGC layer, was thicker in AAV-F-iTrkB-treated GLAST KO mice than in control mice (Figure 4C). Moreover, we examined retinal responses of the second-order kernel, which is impaired in GLAST KO mice^16^^,^^17^ and glaucoma patients,^19^ using multifocal electroretinography (mfERG). We found that retinal responses were higher in AAV-F-iTrkB-treated mice than in control mice (Figure 4D). These data indicate that intraocular injection of AAV-F-iTrkB protects RGCs from death and mitigates retinal degeneration and functional decline in GLAST KO mice.

Next, we examined the therapeutic effects of AAV-F-iTrkB in a mouse model of high-IOP glaucoma. To induce high IOP, we injected silicone oil into the anterior chamber of WT mice to prevent aqueous humor outflow.^20^ Consistent with the previous report, IOP was elevated chronically by 1 week after the silicone oil injection (Figure 4E). In this model, AAV-F-iTrkB significantly increased the number of surviving RGCs compared with the control group (Figure 4F). Due to silicone oil interference, we were unable to obtain reliable data from OCT and mfERG. Collectively, these results suggest that AAV-F-iTrkB could be effective in preventing or slowing the progression of glaucoma associated with both high and normal IOP.

### F-iTrkB protects RGCs following optic nerve injury

**Figure 5:**
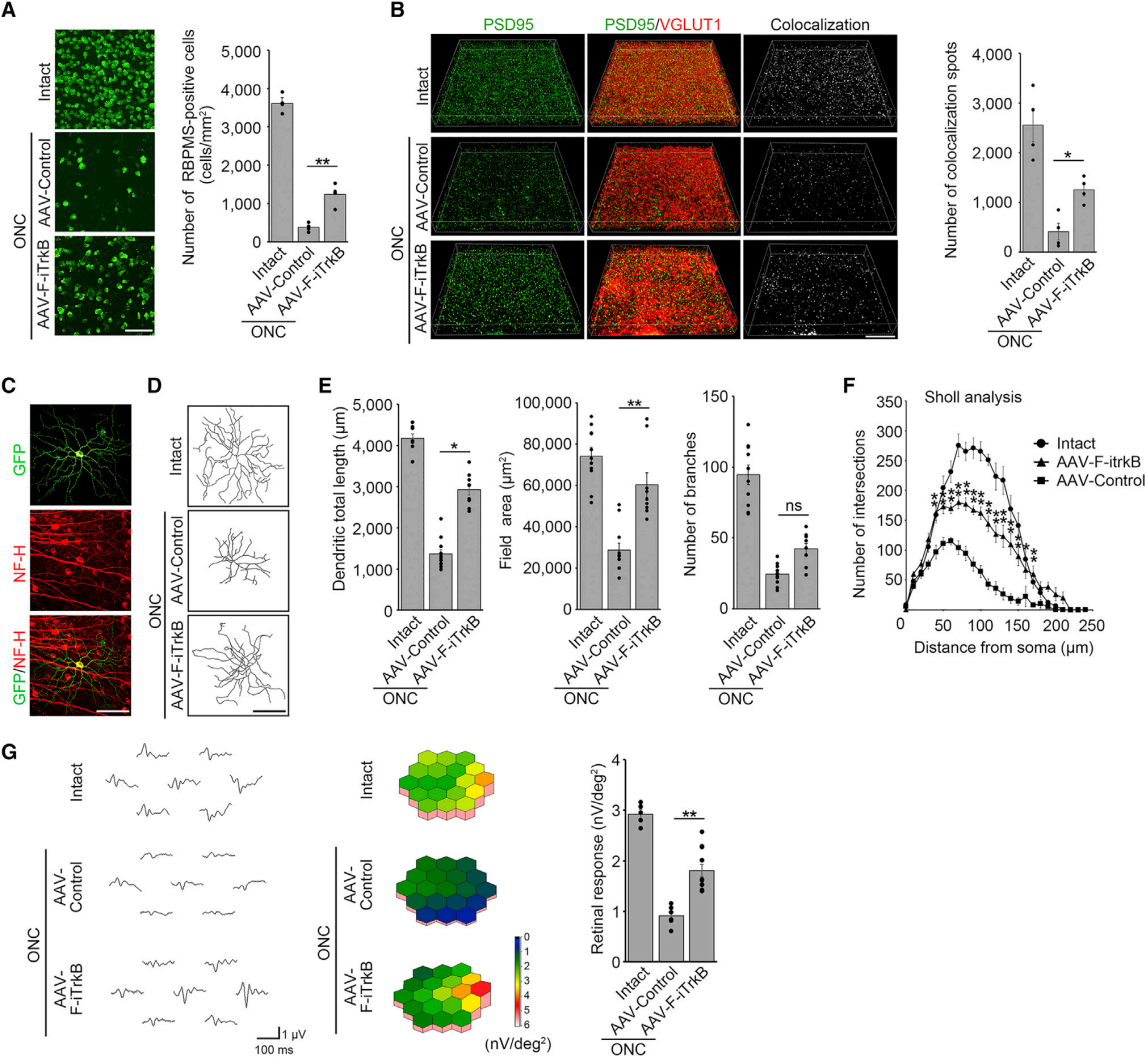
AAV-F-iTrkB prevents RGC degeneration in optic nerve crush injury model (A) F-iTrkB-mediated RGC protection following optic nerve crush (ONC). RGCs were detected by immunostaining of RBPMS at 2 weeks after ONC. Representative images (left) and quantification of the RGC number (right) in intact or injured retinas treated with AAV-Control or AAV-F-iTrkB are shown. The one-way ANOVA with Tukey-Kramer *post hoc* test was used. n = 4 mice per group. ∗∗p < 0.01. Scale bar, 100 μm. (B) F-iTrkB-mediated synapse protection after ONC. Glutamatergic synapses were visualized in flat-mounted retinas using antibodies against PSD95 and VGLUT1, post- and presynaptic markers, respectively. Representative images (left) and quantitative analysis of pre- and postsynaptic colocalized spots (right) in intact or injured retinas treated with AAV-Control or AAV-F-iTrkB are shown. The one-way ANOVA with Tukey-Kramer *post hoc* test was used. n = 4 mice per group. ∗p < 0.05. Scale bar, 30 μm. (C) Representative images of αRGC morphology. αRGC in the retinal flat mount was double labeled with AAV-GFP and an anti-neurofilament-H (NF-H) antibody. Scale bar, 50 μm. (D) Representative images of αRGC dendritic arbors from intact or injured retinas treated with AAV-Control or AAV-F-iTrkB. Scale bar, 50 μm. (E) Quantitative analysis of the dendrite length (left), field area (middle), and number of branches (right) in αRGCs from intact or injured retinas treated with AAV-Control or AAV-F-iTrkB. The one-way ANOVA with Tukey-Kramer *post hoc* test was used. n = 9–11 cells per group. ∗p < 0.05, ∗∗p < 0.01; ns, not statistically significant. (F) Sholl analysis of dendrite morphology as a function of distance from the cell soma in intact or injured retinas treated with AAV-Control or AAV-F-iTrkB. The one-way ANOVA with Tukey-Kramer *post hoc* test was used. n = 9–10 cells per group. ∗p < 0.05, ∗∗p < 0.01. (G) Retinal responses of mice with intact or injured retinas treated with AAV-Control or AAV-F-iTrkB, measured by mfERG. Retinal responses of second-order kernel (left), 3D plot images (middle), and quantitative analyses of the retinal response amplitude (right) are shown. The one-way ANOVA with Tukey-Kramer *post hoc* test was used. n = 5–12 per group. ∗∗p < 0.01.

Furthermore, we examined the effects of AAV-F-iTrkB on RGCs in an acute injury model, the optic nerve crush (ONC) model. ONC was performed 2 weeks after intravitreal administration of AAV-F-iTrkB, and the number of RGCs was counted in retinal flat-mount preparations. Two weeks after ONC, there was a greater number of RGCs in AAV-F-iTrkB-treated mice than in control mice (Figure 5A). These data indicated that AAV-F-iTrkB was also effective in protecting RGCs in an acute retinal degeneration model. Consistent with the reduced number of RGCs, we discovered that synaptic connections, visualized by immunostaining with PSD95 and VGLUT1, also decreased in the control group, but the extent of reduction was milder in AAV-F-iTrkB-treated mice (Figure 5B). The reduced number of synaptic connections could be attributed to cell death and dendritic degeneration. Therefore, we examined the effects of AAV-F-iTrkB on RGC dendritic degeneration. Dendrite morphology was visualized by labeling RGCs with AAV-GFP. We focused on αRGC dendrites rather than dying RGCs because αRGCs are known to be resistant to ONC-mediated cell death.^21^ RGC dendrites were double labeled with AAV-GFP and anti-neurofilament-H (NF-H) antibody, a marker for αRGCs (Figure 5C).^21^ Three weeks after ONC, dendrite retraction was observed in αRGCs of the control group, but the extent of retraction was less in αRGCs of the AAV-F-iTrkB-treated group (Figure 5D). The total dendritic length, area, and number of branches significantly decreased after ONC, but AAV-F-iTrkB treatment alleviated these effects (Figure 5E). Sholl analysis revealed that the number of intersections following ONC was significantly higher in AAV-F-iTrkB-treated dendrites compared with control dendrites (Figure 5F). These findings indicate that AAV-F-iTrkB treatment minimizes ONC-induced retraction of αRGC dendrites. Accordingly, retinal responses were higher in the AAV-F-iTrkB-treated group than in the AAV-Control-treated group after ONC (Figure 5G). These data indicate that AAV-F-iTrkB protects RGC dendrites and synaptic connections after ONC.

### F-iTrkB promotes RGC axon regeneration in an ONC model

**Figure 6:**
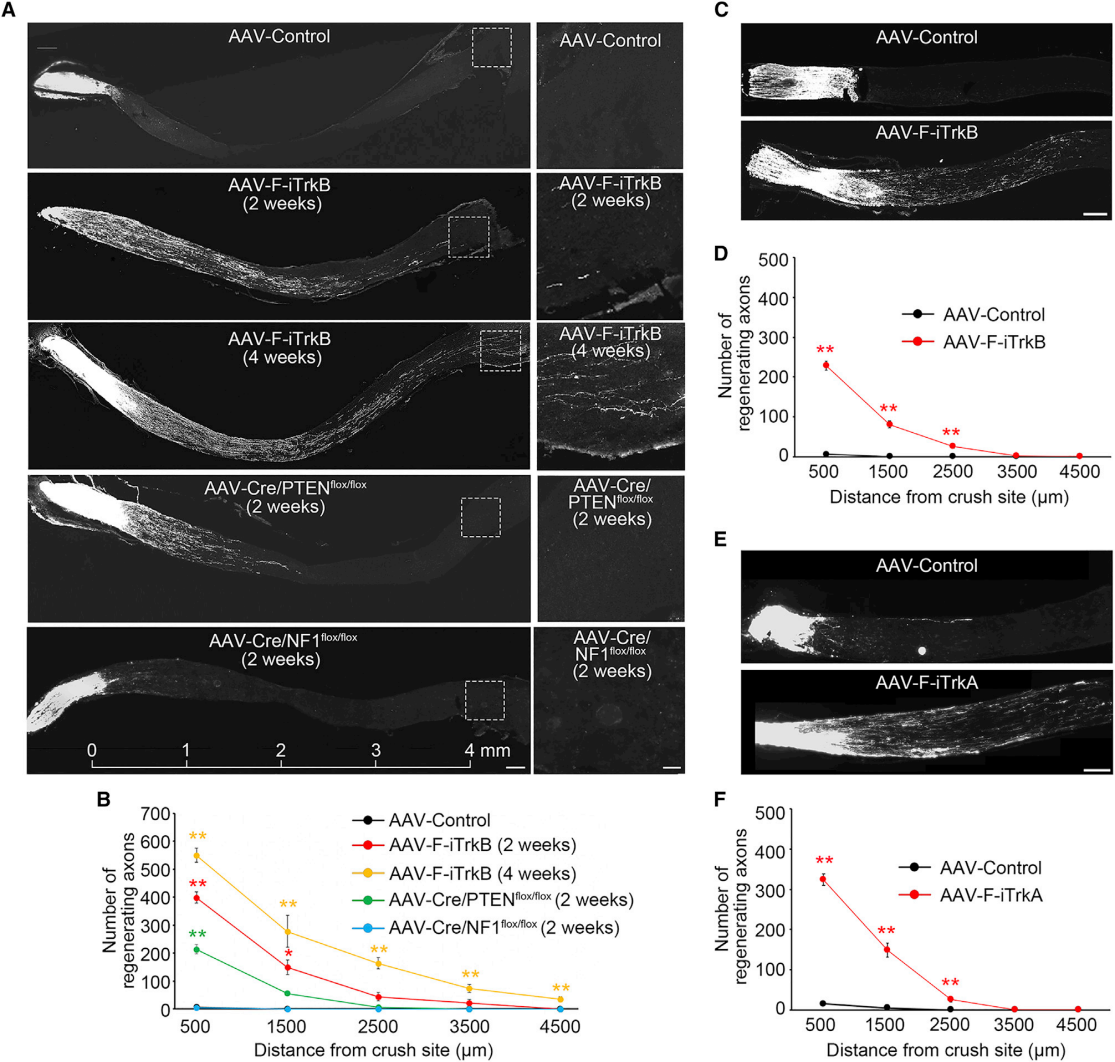
AAV-F-iTrkB promotes robust optic nerve regeneration (A) Representative images of optic nerve sections demonstrating CTB647-labeled regenerating axons in WT mice treated with AAV-F-iTrkB at 2 and 4 weeks after ONC and in PTEN KO and NF1 KO mice at 2 weeks after ONC (left). Scale bar, 200 μm. Magnification of the boxed areas is shown on the right. Scale bar, 50 μm.(B) Quantification of regenerating axons in the optic nerve shown in (A). The one-way ANOVA with Tukey-Kramer *post hoc* test was used. n = 4 mice per group. ∗∗p < 0.01. (C) Representative images of optic nerve sections demonstrating CTB647-labeled regenerating axons in mice that received AAV-F-iTrkB injec tion intravitreally at 3 min after ONC. Scale bar, 200 μm. (D) Quantification of regenerating axons in the optic nerve shown in (C). Two-tailed unpaired Student’s t test was used. n = 4 mice per group. ∗∗p < 0.01. (E) Representative images of optic nerve sections demonstrating CTB647-labeled regenerating axons in mice treated with AAV-F-iTrkA at 2 weeks after ONC. Scale bar, 200 μm. (F) Quantification of regenerating axons in the optic nerve shown in (E). Two-tailed unpaired Student’s t test was used. n = 4 mice per group. ∗∗p < 0.01.

Next, we examined the effects of AAV-F-iTrkB on RGC axon regeneration. Two weeks after AAV injection, ONC was performed, and Alexa Fluor 647-labeled cholera toxin subunit B (CTB647)-labeled regenerated RGC axons were analyzed. A larger volume of CTB647-labeled regenerated RGC axons was observed in the AAV-F-iTrkB-treated group 2 weeks after ONC, and this effect was even greater 4 weeks after ONC (Figure 6A). Four weeks after ONC, some of the regenerated axons reached the optic chiasm, which was approximately 4 mm away from the crush site (Figures 6A and 6B). TrkB activation is widely known to induce downstream signaling pathways, including the PI3K-AKT and Ras-ERK pathways, which are inhibited by PTEN and NF1, respectively. Previous studies have revealed that PTEN deletion induced optic nerve regeneration after ONC,^22^ but the effects of NF1 deletion are unknown. Therefore, we induced PTEN or NF1 deletion by injecting AAV-Cre into the eyes of PTEN^flox/flox^ or NF1^flox/flox^ mice, respectively, 2 weeks before ONC. We discovered that AAV-F-iTrkB treatment resulted in greater RGC axon regeneration than PTEN deletion (Figures 6A and 6B). These data indicate that F-iTrkB is more powerful than unleashing endogenous levels of PI3K signaling by removing its suppressor PTEN. However, NF1, a Ras GTPase activating protein (Ras-GAP) that suppresses Ras signaling, had no effects on RGC axon regeneration (Figures 6A and 6B), indicating that NF1 is not a major Ras-GAP in RGCs.

We injected AAV-F-iTrkB 3 min after ONC to assess its potential for clinical use. AAV-F-iTrkB significantly stimulated the optic nerve regeneration compared with AAV-Control, although the effect was not as significant as that of preadministration, and it was similar to that of PTEN deletion (Figures 6C and 6D). We also examined the ability of AAV-F-iTrkA to stimulate RGC axon regeneration because F-iTrkA strongly activated its downstream signaling pathways *in vitro* (Figure 2G). Intravitreal administration of AAV-F-iTrkA promoted RGC axon regeneration to a similar level compared with AAV-F-iTrkB (Figures 6E and 6F), indicating that AAV-F-iTrkA is as effective as AAV-F-iTrkB. Collectively, these data demonstrate that AAV-F-iTrkB treatment can protect neurons from disease and injury and mediate robust axon regeneration.

### F-iTrkB promotes RGC axon regeneration in an optic tract transection model

**Figure 7:**
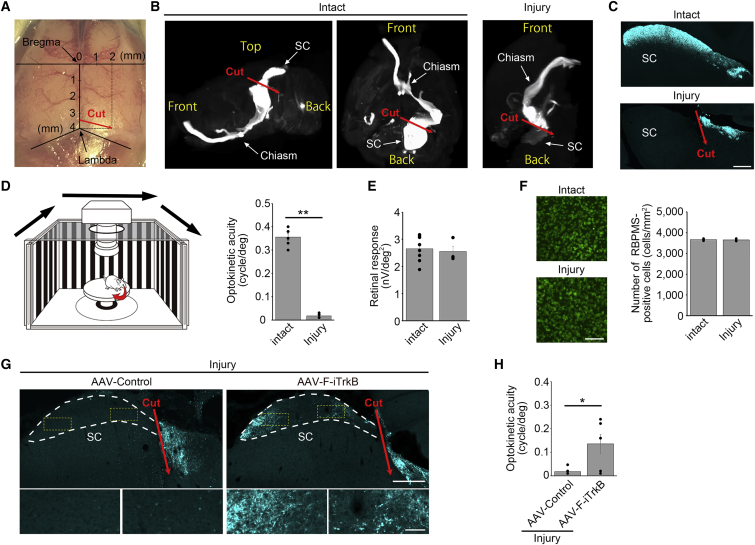
AAV-F-iTrkB promotes axon regeneration in an optic tract transection model (A) A photograph indicating the position of the surgical incision for optic tract transection. (B) Representative images of the optic tract traced with CTB647 in the intact brain and in the brain that received optic tract transection (injured brain). CTB647 was injected intravitreally and the optic tract from the eye to the superior colliculus (SC) in the brain was made visible by a tissue-clearing technique. (C) Representative images of sagittal brain sections demonstrating CTB647-labeled axons near the SC in intact and injured brains. Scale bar, 300 μm. (D) Measurement of optokinetic responses to evaluate visual behavior (left). Optokinetic acuity of intact mice or mice with optic tract transection (injured mice) is shown (right). Two-tailed unpaired Student’s t test was used. n = 4–6 mice per group. ∗∗p < 0.01. (E) Averaged retinal responses of second-order kernel measured by mfERG in intact and injured mice. n = 4–8 mice per group. (F) Representative images of retinal flat mounts immunostained with an anti-RBPMS antibody in intact and injured mice at 12 weeks after injury (left). Scale bar, 100 μm. Quantification of the number of RBPMS-positive cells (right). n = 4–5 mice per group. (G) Representative images of sagittal brain sections demonstrating CTB647-labeled regenerating axons near the SC in mice treated with AAV-Control or AAV-F-iTrkB (top). The SC areas were marked with white dotted lines. Scale bar, 300 μm. Magnifications of the areas boxed in yellow are shown at the bottom. Scale bar, 50 μm. (H) Optokinetic acuity of intact or injured mice treated with AAV-Control or AAV-F-iTrkB. Two-tailed unpaired Student’s t test was used. n = 6 mice per group. ∗p < 0.05.

As shown above, F-iTrkB induced robust axon regeneration; however, regenerated axons that reached the optic chiasm were sparse, making it difficult to determine if F-iTrkB could repair the visual pathway effectively after injury. Hence, we employed an optic tract transection model in which the distance between the injury site and the axonal projection site was significantly shorter than in the ONC model. For this, RGC axons in adult mice were cut near the SC (Figure 7A). The CTB647-labeled optic tract is shown in a 3D image via tissue clearing (Figure 7B) and in frozen sections (Figure 7C). In the injured mice, the optokinetic responses (OKRs) were lost 10 weeks after injury (Figure 7D). Unlike the ONC model, optic tract transection did not induce retinal function loss (Figure 7E) or RGC death (Figure 7F) for at least 12 weeks after injury. In this model, no CTB647-labeled axons were detected in the SC of control mice, but CTB647-labeled axons were observed in the SC of AAV-F-iTrkB-treated mice (Figure 7G). We measured OKRs 10–12 weeks after injury and discovered that optokinetic acuity was slightly higher in the AAV-F-iTrkB-treated group compared with the control group (Figure 7H). These data indicate that AAV-F-iTrkB can promote RGC axon regeneration even when the injury site is far from the cell body.

## Discussion

In this study, we developed a gene therapy tool that enhances TrkB signaling in neurons in the absence of ligands. Intraocular AAV-F-iTrkB administration changed the expression of numerous genes in RGCs and suppressed RGC death in two mouse models of glaucoma and after ONC. Recent studies have revealed that increased TrkB signaling in RGCs could be an effective therapy for glaucoma, but maintaining a continued supply of BDNF is impractical.^14^^,^^23^^,^^24^ To solve this problem, there is a gene therapy construct, TrkB-2A-mBDNF, designed to achieve sustained BDNF-TrkB signaling,^25^ and AAV2-TrkB-2A-mBDNF has neuroprotective efficacy in a mouse ONC model.^26^ This is a unique system, but we cannot directly compare its effect with those of F-iTrkB because the construction of the vector, especially the promoter region, is different with our system. In addition to gene therapies, TrkB agonists are showing positive results in promoting RGC survival in acute and chronic models of glaucoma.^27^^,^^28^ It is reported that treatment with 7,8-DHF, an agonist of the TrkB receptor, leads to upregulation in the tyrosine phosphorylation on TrkB residues, activates its downstream signaling, and enhances RGC survival.^29^^,^^30^ Deoxygedunin is another compound that is a potent and specific agonist of TrkB and has been shown to be neuroprotective.^31^ It will be interesting to compare the effects of TrkB agonists with TrkB gene therapies to develop more efficient and safer therapeutic strategies in the future.

Adult CNS axons do not usually regenerate after injury, but recent studies indicate that genetic manipulation can put neurons into a regenerative state, implying that CNS axon regeneration is possible.^32^ A literature review indicates that the discovery of the effects of PTEN deletion on axon regeneration was a breakthrough in this field,^22^ and subsequent studies attempted to achieve greater length and intensity of axon regeneration primarily using combinatory approaches, such as PTEN deletion with manipulation of other genes, including SOCS3,^33^ Lin28,^34^ and ATF3.^35^ We discovered that intraocular injection of AAV-F-iTrkB alone into WT mice induced robust axon regeneration, with some axons reaching the optic chiasm after ONC. Such a high degree of regeneration by gene manipulation is remarkable, and although a direct comparison of efficacy across different approaches is difficult due to differences in experimental settings, we believe that the regenerative ability of AAV-F-iTrkB competes with the best available at present.

Additional studies are required to further stimulate optic nerve regeneration and recover visual function. One possible approach is the combination of AAV-F-iTrkB with other strategies. Currently, the long-term delivery of CNTF by an intravitreal implant with encapsulated cells secreting CNTF is in phase II clinical trials for glaucoma (ClinicalTrials.gov; identifier: NCT02862938). CNTF promotes RGC axon regeneration and protects RGCs.^6^ Since F-igp130 and F-iLIFR failed to activate CNTF receptor signaling, combinatory treatment with a CNTF implant and AAV-F-iTrkB injection could produce synergistic effects. There was a concern that constitutive activity of TrkB could result in tumor growth, but the intravitreal administration of AAV-F-iTrkB did not have abnormal cell growth or negative side effects. Future studies will investigate the use of inducible systems, such as a light-switchable transgene system and a tamoxifen-inducible Cre/*loxP* system. For long-term observation, we intend to examine the effectiveness of AAV-F-iTrkB gene therapy in marmoset models of ONC and glaucoma.^23^ The powerful axon regeneration by F-iTrkB encourages the idea that iTrkB may also be effective in treating spinal cord injury. It has been observed that the expression of truncated forms of TrkB (without the intracellular catalytic tyrosine kinase domain) is significantly increased after spinal cord injury, suggesting that this increased expression could limit the availability of BDNF to facilitate axon regeneration.^36^ Since AAV-F-iTrkB can stimulate intracellular signaling without ligands, F-iTrkB may induce axon regeneration regardless of the increased expression of truncated forms of TrkB.

Conclusively, intraocular injection of AAV-F-iTrkB protected RGCs in animal models of glaucoma and induced robust axon regeneration after axon injury (Figure S2). The powerful therapeutic effect is a great advantage because it may eliminate tissue damage caused by frequent injections. With further characterization and enhancement of delivery, AAV-F-iTrkB may become an effective gene therapy tool for axonal damage and some neurodegenerative diseases, including glaucoma.

## Materials and methods

### Animals

Experiments were performed using C57BL/6J, RiboTag,^15^ TrkB^flox/flox^;c-kit-Cre (TrkB^c-kit^ KO),^12^ GLAST KO,^17^ PTEN^flox/flox^, and NF1^flox/flox^ mice.^37^ RiboTag and PTEN^flox/flox^ mice were purchased from The Jackson Laboratory. The animals were treated in accordance with the Tokyo Metropolitan Institute of Medical Science *Guidelines for the Care and Use of Animals*. All animal experiments were approved by the Institutional Animal Care and Use Committee of the Tokyo Metropolitan Institute of Medical Science (18041).

### Plasmids

A plasmid encoding human TrkB was purchased from OriGene Technologies. TrkB mutants were generated by site-directed mutagenesis. The farnesylation signal of K-Ras or myristoylation signal of annexin was used for membrane localization.^38^^,^^39^^,^^40^ GRB2, Shc, PLC, TrkA, gp130, and LIFR constructs were obtained from mouse brain cDNA by PCR.

### Transfection and immunoblot analysis

Transient transfection in Cos-7 cells or Neuro2A cells was performed using Polyethylenimine HCl Max (Polyscience). After transfection for 20 h, the cells were lysed in SDS-PAGE loading buffer. For signal transduction analysis, cells were serum starved for 1.5 h before cell lysis. The samples were subjected to immunoblot analysis using antibodies listed in Table S1. Quantitative analysis was carried out using ImageJ version 2.0.0.^41^

### Pull-down assay

Cos-7 cells transfected with plasmids of interest were lysed with a lysis buffer (25 mM Tris [pH 7.4], 150 mM NaCl, 1% Triton X-100) and centrifuged at 16,000 × *g* for 10 min. The supernatant was incubated with glutathione Sepharose 4B resin (Thermo Fisher Scientific) for 30 min at 4°C with gentle agitation. After washing, the precipitated samples were subjected to immunoblot analysis.

### Preparation of AAV

AAVs were produced and purified as described previously.^42^^,^^43^ Briefly, HEK293 cells were transiently transfected with the AAV vector, pRC2-mi342, and pHelper plasmids (TAKARA). Seventy-two hours after transfection using Polyethylenimine HCl Max, the cells were harvested by scraping, followed by three cycles of freeze-thawing. Cell debris was pelleted at 5,000 × *g* for 20 min, and supernatants were treated with Benzonase (200 U/mL; Merck) in the presence of 5 mM MgCl_2_ at 37°C for 1 h. The Benzonase-treated viral solution was run on an iodixanol gradient. Purified AAV vectors were washed with Hanks’ balanced salt solution (HBSS) and concentrated using a VIVASPIN 20, 100 kDa MWCO (Sartorius Stedim Lab). Virus titers were determined by quantitative PCR.

### Purification of RGC-specific RNA

RiboTag mice were injected intravitreally with AAV-Cre for labeling of RGCs. Subsequently, AAV-F-iTrkB was injected intravitreally. Purification of RGC ribosomes was performed as described previously,^44^^,^^45^ with minor modifications. The mice were perfused transcardially with ice-cold phosphate-buffered saline (PBS), and retinas were dissected. Six retinas were pooled as one sample and were homogenized with a Dounce homogenizer in a homogenization buffer (50 mM Tris-HCl [pH 7.5], 100 mM KCl, 12 mM MgCl_2_, 1% Nonidet P-40, 1 mM DTT, 200 U/mL RNasin, 100 μg/mL cycloheximide, 1 mg/mL heparin). The homogenates were centrifuged at 15,000 × *g* at 4°C for 15 min. The supernatant was incubated with a mouse monoclonal anti-HA antibody (1:50; Biolegend) at 4°C for 16 h. Ribosomes bound to an anti-HA antibody were purified using protein G magnetic beads (GE Healthcare). The RNA bound to ribosomes was purified with an RNeasy Protect Mini Kit (QIAGEN) according to the manufacturer’s instructions.

### RNA sequencing and analysis

Sequencing and analysis of the purified RNA were performed by the Novogene NGS Analysis Service (Novogene). Briefly, sequencing libraries were generated using a NEBNext Ultra RNA Library Prep Kit for Illumina (New England Biolabs) and index codes were added to attribute sequences to each sample. Clustering of the index-coded samples was performed on a cBot Cluster Generation System using a PE Cluster Kit cBot-HS (Illumina). After cluster generation, the library preparations were sequenced on an Illumina platform and 125-bp/150-bp paired-end reads were generated.

An index of the reference genome was built using Bowtie v.2.2.3^46^ and paired-end clean reads were aligned to the reference genome using TopHat v.2.0.12.^47^ HTSeq v.0.6.1^48^ was used to count the read numbers mapped to each gene. Differential expression analysis of two conditions/group (two biological replicates per condition) was performed using the DESeq R package 1.18.0.^49^ The p values were adjusted using the Benjamini-Hochberg method. An adjusted p of 0.005 and log2(fold change) of 1 were set as the threshold for significantly different expression. GO analysis was performed separately for up- and downregulated gene lists using DAVID Bioinformatics Resources 6.8 (https://david.ncifcrf.gov). KOBAS^50^ was used to test the statistical enrichment of differentially expressed genes in KEGG pathways.

### Immunostaining of retinal flat mounts and quantification of RGC number

Mice were perfused with 4% paraformaldehyde (PFA), and the eyes were enucleated. The removed retinas were first incubated for 2 h in a blocking solution containing 5% horse serum and 1% Triton X-100 in PBS (pH 7.4). The retinas were then incubated for 24 h with primary antibodies, anti-pERK antibody (1:1,000; Cell Signaling), pAKT antibody (1:1,000; Cell Signaling), or anti-RBPMS antibody (1:1,000; MERCK), followed by incubation with fluorescence-labeled secondary antibodies (Table S1) at room temperature for 2 h. Images were obtained using the FV3000 confocal microscope (Olympus) or All-in-One fluorescence microscope BZ-X800 (Keyence). The number of RBPMS-positive RGCs was obtained from one central (0.1 mm from the optic disc), one middle (0.8 mm from the optic disc), and one peripheral (1.5 mm from the optic disc) area (0.04 mm^2^) per quadrant (dorsal, ventral, nasal, and temporal parts) of each retina.^18^ The average density of RGCs per square millimeter was calculated.

### Retrograde labeling of RGCs

Retrograde labeling of RGCs was conducted as described previously.^51^ Briefly, 1% Fluoro-gold (Fluorochrome) dissolved in PBS was injected into the SC using a microsyringe. At 10 days after injection, the mice were sacrificed, and the eyes were enucleated. The retinas were flat mounted on microscope slides for examination under a BZ-X800 fluorescence microscope (Keyence).

### OCT imaging

OCT (RS-3000; Nidek) imaging was performed as described previously.^17^ All line-scan images were taken at a distance of three-disc diameters from the optic disc and the average thickness of the GCC (from the inner limiting membrane to the outer boundary of the inner plexiform layer) was measured in retinal images obtained by circular scanning around the optic disc.

### mfERG

mfERG was performed using a VERIS 6.0 system (Electro-Diagnostic Imaging) as described previously.^16^^,^^17^ Briefly, the visual stimulus consisted of seven hexagonal areas scaled with eccentricity. The stimulus array was displayed on a high-resolution black-and-white monitor driven at a frame rate of 100 Hz. The second-order kernel was analyzed as reported previously.^16^^,^^17^

### Induction of IOP elevation by intracameral injection of silicone oil

Injection of silicone oil was performed as described previously.^20^ Briefly, mice were anesthetized with an intraperitoneal injection of a mixture of medetomidine, midazolam, and butorphanol. A 33G needle was tunneled through the layers of the cornea to reach the anterior chamber without injuring the lens or iris. Silicone oil (1,000 mPa s; Alfa Aesar) was injected slowly into the anterior chamber until the oil droplet expanded to cover most areas of the iris.

### ONC

The mice were anesthetized with isoflurane during ONC. The optic nerve was exposed intraorbitally and crushed for 5 s at 0.5 mm from the posterior pole of the eyeball with fine surgical forceps.^38^^,^^52^

### RGC dendritic arbor imaging

AAV-GFP was administered intravitreally at 2.0 × 10^9^ vector genomes/retina so that the neighboring RGC dendrites did not overlap with one another. At 2 weeks after AAV-GFP injection, the mice were perfused with 4% PFA, and retinas were immunostained with an anti-NF-H antibody (SMI32 clone, 1:1,000; Biolegend) for detection of αRGCs. To visualize GFP-labeled dendrites clearly, the immunostained retinal flat mounts were immersed in 85% glycerol^52^ and imaged with an FV3000 confocal microscope (Olympus). The obtained z-stack images were reconstituted as 3D images using Imaris software v.9.2.1 (Bitplane). GFP-labeled dendrites were traced using the Imaris filament tracing function.

### Quantification of RGC synapses

To visualize RGC synapses in the inner retinal layer, retinal flat mounts were incubated with anti-BNPI (VGLUT1, 1:1,000; Santa Cruz) and anti-PSD95 (1:1,000; Cell Signaling) antibodies for 48 h at 4°C with gentle agitation, followed by incubation with fluorescence-labeled secondary antibodies (Table S1). Images were obtained from the middle region (0.8 mm from the optic disc) with an FV3000 confocal microscope (Olympus). To reconstruct high-resolution 3D images, scans were taken at 0.5-μm intervals with 30 images per focal plane using a 100× objective lens. The number of double-immunolabeled synapses was counted in a volume of 100 × 100 × 15 μm of each retina using ImarisColoc analysis.

### Quantification of regenerating axons

To visualize regenerating axons in the optic nerve, 2 μL of CTB647 (Thermo Fisher) was injected intravitreally at 2 days before sacrifice. Frozen sections of the optic nerves (14 μm thickness) were obtained by cryosectioning, and CTB647-positive axons were counted manually at 500, 1,500, 2,500, 3,500, and 4,500 μm distal to the lesion site. The total number of regenerating axons at different sites in the optic nerve was calculated from the obtained data.^38^

### Optic tract transection

Optic tract transection was conducted as described previously.^53^ Briefly, the mice were anesthetized, shaved, disinfected, and placed in a stereotaxic apparatus. A small incision was made on the scalp and a bone flap was created over the SC. The optic tract was cut with a sharp knife at 3.5 mm posterior to the bregma on the midline to 4 mm posterior to the bregma and 2 mm lateral to the midline at a depth of 2.5 mm (Figure 7A). An antibacterial ointment was applied and the skin was sutured with a 6-0 silk thread.

### 3D visualization of the visual pathway

To visualize the visual pathway in the mouse brain, 2 μL of CTB647 was injected intravitreally. At 2 days after injection, the mice were perfused transcardially with PBS followed by a fixation buffer (4% PFA in 0.1 M phosphate buffer [pH 7.4]). The whole brain was dissected out and postfixed with a fixation buffer overnight at 4°C. Tissue clearing was performed by using the 3DISCO method with some modifications.^54^ Briefly, the brain was incubated in 50% tetrahydrofuran (THF) in distilled water for 12 h, 70% THF for 12 h, 80% THF for 12 h, 100% THF for 3 × 12 h, and dibenzyl ether for 2–3 h before imaging. The cleared brain was immersed in dibenzyl ether (refractive index 1.562) and imaged with a light sheet fluorescence microscope (MVX10-LS; Olympus).^55^ We used a 2× objective lens with a 640-nm laser and bandpass filter (660/750 nm). Approximately 500 images were collected by scanning the sample in the z direction with an 8-μm step size. The obtained z*-*stack images were reconstituted as 3D images using Imaris software (v.9.2.1).

### Visual behavior test

OKRs were analyzed using OptoMotry (Cerebral Mechanics) to measure the highest spatial frequency of the grating tracked, as described previously.^53^ The mice were placed on an elevated platform surrounded by four computer monitors displaying black-and-white bars. If the head of the mouse moved in concert with the gratings, the trial was scored as “tracked.” The optokinetic acuity was determined using an automated staircase procedure. The injured eye refers to the left eye contralateral to the injured right SC. The test normally lasted for 5 min.

### Statistical analysis

Statistics were performed using JMP 15.2.0 software (SAS Institute). Data are presented as the mean ± SEM. Data significance was determined using two-tailed Student’s t tests or one-way ANOVA with Tukey-Kramer *post hoc* test. Statistical significance is reported as significant at p < 0.05.

## Supplemental information

Document S1. Figures S1 and S2 and Table S1

Document S2. Article plus supplemental information

## References

[1] Malihi M., Moura Filho E.R., Hodge D.O., Sit A.J. (2014). Long-term trends in glaucoma-related blindness in Olmsted County, Minnesota. Ophthalmology.

[2] Yokoyama Y., Maruyama K., Konno H., Hashimoto S., Takahashi M., Kayaba H., Kokubun T., Nakazawa T. (2015). Characteristics of patients with primary open angle glaucoma and normal tension glaucoma at a university hospital: a cross-sectional retrospective study. BMC Res. Notes.

[3] O'Connor D.M., Boulis N.M. (2015). Gene therapy for neurodegenerative diseases. Trends Mol. Med..

[4] Wilson A.M., Di Polo A. (2012). Gene therapy for retinal ganglion cell neuroprotection in glaucoma. Gene Ther..

[5] Ren R., Li Y., Liu Z., Liu K., He S. (2012). Long-term rescue of rat retinal ganglion cells and visual function by AAV-mediated BDNF expression after acute elevation of intraocular pressure. Invest. Ophthalmol. Vis. Sci..

[6] Leaver S.G., Cui Q., Plant G.W., Arulpragasam A., Hisheh S., Verhaagen J., Harvey A.R. (2006). AAV-mediated expression of CNTF promotes long-term survival and regeneration of adult rat retinal ganglion cells. Gene Ther..

[7] Pernet V., Di Polo A. (2006). Synergistic action of brain-derived neurotrophic factor and lens injury promotes retinal ganglion cell survival, but leads to optic nerve dystrophy in vivo. Brain..

[8] Dewitt J., Ochoa V., Urschitz J., Elston M., Moisyadi S., Nishi R. (2014). Constitutively active TrkB confers an aggressive transformed phenotype to a neural crest-derived cell line. Oncogene.

[9] Lerner E.C., Qian Y., Blaskovich M.A., Fossum R.D., Vogt A., Sun J., Cox A.D., Der C.J., Hamilton A.D., Sebti S.M. (1995). Ras CAAX peptidomimetic FTI-277 selectively blocks oncogenic Ras signaling by inducing cytoplasmic accumulation of inactive Ras-Raf complexes. J. Biol. Chem..

[10] Wright L.P., Philips M.R. (2006). Thematic review series: lipid posttranslational modifications. CAAX modification and membrane targeting of Ras. J. Lipid Res..

[11] Harada T., Harada C., Parada L.F. (2007). Molecular regulation of visual system development: more than meets the eye. Genes Dev..

[12] Harada C., Guo X., Namekata K., Kimura A., Nakamura K., Tanaka K., Parada L.F., Harada T. (2011). Glia- and neuron-specific functions of TrkB signalling during retinal degeneration and regeneration. Nat. Commun..

[13] Kimura A., Namekata K., Guo X., Harada C., Harada T. (2016). Neuroprotection, growth factors and BDNF-TrkB signalling in retinal degeneration. Int. J. Mol. Sci..

[14] Gupta V., You Y., Li J., Gupta V., Golzan M., Klistorner A., van den Buuse M., Graham S. (2014). BDNF impairment is associated with age-related changes in the inner retina and exacerbates experimental glaucoma. Biochim. Biophys. Acta.

[15] Sanz E., Yang L., Su T., Morris D.R., McKnight G.S., Amieux P.S. (2009). Cell-type-specific isolation of ribosome-associated mRNA from complex tissues. Proc. Natl. Acad. Sci. USA.

[16] Harada T., Harada C., Nakamura K., Quah H.M.A., Okumura A., Namekata K., Saeki T., Aihara M., Yoshida H., Mitani A., Tanaka K. (2007). The potential role of glutamate transporters in the pathogenesis of normal tension glaucoma. J. Clin. Invest..

[17] Sano H., Namekata K., Kimura A., Shitara H., Guo X., Harada C., Mitamura Y., Harada T. (2019). Differential effects of N-acetylcysteine on retinal degeneration in two mouse models of normal tension glaucoma. Cell Death Dis..

[18] Honda S., Namekata K., Kimura A., Guo X., Harada C., Murakami A., Matsuda A., Harada T. (2019). Survival of alpha and intrinsically photosensitive retinal ganglion cells in NMDA-induced neurotoxicity and a mouse model of normal tension glaucoma. Invest. Ophthalmol. Vis. Sci..

[19] Sutter E.E., Bearse M.A. (1999). The optic nerve head component of the human ERG. Vis. Res..

[20] Zhang J., Li L., Huang H., Fang F., Webber H.C., Zhuang P., Liu L., Dalal R., Tang P.H., Mahajan V.B. (2019). Silicone oil-induced ocular hypertension and glaucomatous neurodegeneration in mouse. Elife.

[21] Duan X., Qiao M., Bei F., Kim I.J., He Z., Sanes J.R. (2015). Subtype-specific regeneration of retinal ganglion cells following axotomy: effects of osteopontin and mTOR signaling. Neuron.

[22] Park K.K., Liu K., Hu Y., Smith P.D., Wang C., Cai B., Xu B., Connolly L., Kramvis I., Sahin M., He Z. (2008). Promoting axon regeneration in the adult CNS by modulation of the PTEN/mTOR pathway. Science.

[23] Noro T., Namekata K., Kimura A., Azuchi Y., Hashimoto N., Moriya-Ito K., Komaki Y., Lee C.Y., Okahara N., Guo X. (2019). Normal tension glaucoma-like degeneration of the visual system in aged marmosets. Sci. Rep..

[24] Cheng L., Sapieha P., Kittlerova P., Hauswirth W.W., Di Polo A. (2002). TrkB gene transfer protects retinal ganglion cells from axotomy-induced death in vivo. J. Neurosci..

[25] Osborne A., Wang A.X.Z., Tassoni A., Widdowson P.S., Martin K.R. (2018). Design of a novel gene therapy construct to achieve sustained brain-derived neurotrophic factor signaling in neurons. Hum. Gene Ther..

[26] Osborne A., Khatib T.Z., Songra L., Barber A.C., Hall K., Kong G.Y.X., Widdowson P.S., Martin K.R. (2018). Neuroprotection of retinal ganglion cells by a novel gene therapy construct that achieves sustained enhancement of brain-derived neurotrophic factor/tropomyosin-related kinase receptor-B signaling. Cell Death Dis..

[27] Hu Y., Cho S., Goldberg J.L. (2010). Neurotrophic effect of a novel TrkB agonist on retinal ganglion cells. Invest. Ophthalmol. Vis. Sci..

[28] Bai Y., Shi Z., Zhuo Y., Liu J., Malakhov A., Ko E., Burgess K., Schaefer H., Esteban P.F., Tessarollo L., Saragovi H.U. (2010). In glaucoma the upregulated truncated TrkC.T1 receptor isoform in glia causes increased TNF-alpha production, leading to retinal ganglion cell death. Invest. Ophthalmol. Vis. Sci..

[29] Gupta V.K., You Y., Li J.C., Klistorner A., Graham S.L. (2013). Protective effects of 7, 8-dihydroxyflavone on retinal ganglion and RGC-5 cells against excitotoxic and oxidative stress. J. Mol. Neurosci..

[30] Gupta V.K., You Y., Klistorner A., Graham S.L. (2012). Shp-2 regulates the TrkB receptor activity in the retinal ganglion cells under glaucomatous stress. Biochim. Biophys. Acta.

[31] Jang S.W., Liu X., Chan C.B., France S.A., Sayeed I., Tang W., Lin X., Xiao G., Andero R., Chang Q. (2010). Deoxygedunin, a natural product with potent neurotrophic activity in mice. PLoS One.

[32] Williams P.R., Benowitz L.I., Goldberg J.L., He Z. (2020). Axon regeneration in the mammalian optic nerve. Annu. Rev. Vis. Sci..

[33] Sun F., Park K.K., Belin S., Wang D., Lu T., Chen G., Zhang K., Yeung C., Feng G., Yankner B.A., He Z. (2011). Sustained axon regeneration induced by co-deletion of PTEN and SOCS3. Nature.

[34] Wang X.W., Li Q., Liu C.M., Hall P.A., Jiang J.J., Katchis C.D., Kang S., Dong B.C., Li S., Zhou F.Q. (2018). Lin28 signaling supports mammalian PNS and CNS axon regeneration. Cell Rep..

[35] Kole C., Brommer B., Nakaya N., Sengupta M., Bonet-Ponce L., Zhao T., Wang C., Li W., He Z., Tomarev S. (2020). Activating transcription factor 3 (ATF3) protects retinal ganglion cells and promotes functional preservation after optic nerve crush. Invest. Ophthalmol. Vis. Sci..

[36] Liebl D.J., Huang W., Young W., Parada L.F. (2001). Regulation of Trk receptors following contusion of the rat spinal cord. Exp. Neurol..

[37] Zhu Y., Romero M.I., Ghosh P., Ye Z., Charnay P., Rushing E.J., Marth J.D., Parada L.F. (2001). Ablation of NF1 function in neurons induces abnormal development of cerebral cortex and reactive gliosis in the brain. Genes Dev..

[38] Namekata K., Harada C., Taya C., Guo X., Kimura H., Parada L.F., Harada T. (2010). Dock3 induces axonal outgrowth by stimulating membrane recruitment of the WAVE complex. Proc. Natl. Acad. Sci. USA.

[39] Wice B.M., Gordon J.I. (1992). A strategy for isolation of cDNAs encoding proteins affecting human intestinal epithelial cell growth and differentiation: characterization of a novel gut-specific N-myristoylated annexin. J. Cell Biol..

[40] Jiang H., Zhang X., Chen X., Aramsangtienchai P., Tong Z., Lin H. (2018). Protein lipidation: occurrence, mechanisms, biological functions, and enabling technologies. Chem. Rev..

[41] Rueden C.T., Schindelin J., Hiner M.C., DeZonia B.E., Walter A.E., Arena E.T., Eliceiri K.W. (2017). ImageJ2: ImageJ for the next generation of scientific image data. BMC Bioinformatics.

[42] Udagawa T., Fujioka Y., Tanaka M., Honda D., Yokoi S., Riku Y., Ibi D., Nagai T., Yamada K., Watanabe H. (2015). FUS regulates AMPA receptor function and FTLD/ALS-associated behaviour via GluA1 mRNA stabilization. Nat. Commun..

[43] Zolotukhin S., Byrne B.J., Mason E., Zolotukhin I., Potter M., Chesnut K., Summerford C., Samulski R.J., Muzyczka N. (1999). Recombinant adeno-associated virus purification using novel methods improves infectious titer and yield. Gene Ther..

[44] Itoh N., Itoh Y., Tassoni A., Ren E., Kaito M., Ohno A., Ao Y., Farkhondeh V., Johnsonbaugh H., Burda J. (2018). Cell-specific and region-specific transcriptomics in the multiple sclerosis model: focus on astrocytes. Proc. Natl. Acad. Sci. USA.

[45] Guo X., Kimura A., Namekata K., Harada C., Arai N., Takeda K., Ichijo H., Harada T. (2022). ASK1 signaling regulates phase-specific glial interactions during neuroinflammation. Proc. Natl. Acad. Sci. USA.

[46] Langmead B., Salzberg S.L. (2012). Fast gapped-read alignment with Bowtie 2. Nat. Methods.

[47] Kim D., Pertea G., Trapnell C., Pimentel H., Kelley R., Salzberg S.L. (2013). TopHat2: accurate alignment of transcriptomes in the presence of insertions, deletions and gene fusions. Genome Biol..

[48] Anders S., Pyl P.T., Huber W. (2015). HTSeq--a Python framework to work with high-throughput sequencing data. Bioinformatics.

[49] Anders S., Huber W. (2010). Differential expression analysis for sequence count data. Genome Biol..

[50] Mao X., Cai T., Olyarchuk J.G., Wei L. (2005). Automated genome annotation and pathway identification using the KEGG Orthology (KO) as a controlled vocabulary. Bioinformatics.

[51] Azuchi Y., Namekata K., Shimada T., Guo X., Kimura A., Harada C., Saito A., Yamagata K., Harada T. (2018). Role of neuritin in retinal ganglion cell death in adult mice following optic nerve injury. Sci. Rep..

[52] Namekata K., Guo X., Kimura A., Arai N., Harada C., Harada T. (2019). DOCK8 is expressed in microglia, and it regulates microglial activity during neurodegeneration in murine disease models. J. Biol. Chem..

[53] Bei F., Lee H.H.C., Liu X., Gunner G., Jin H., Ma L., Wang C., Hou L., Hensch T.K., Frank E. (2016). Restoration of visual function by enhancing conduction in regenerated axons. Cell.

[54] Ertürk A., Becker K., Jährling N., Mauch C.P., Hojer C.D., Egen J.G., Hellal F., Bradke F., Sheng M., Dodt H.U. (2012). Three-dimensional imaging of solvent-cleared organs using 3DISCO. Nat. Protoc..

[55] Luo X., Salgueiro Y., Beckerman S.R., Lemmon V.P., Tsoulfas P., Park K.K. (2013). Three-dimensional evaluation of retinal ganglion cell axon regeneration and pathfinding in whole mouse tissue after injury. Exp. Neurol..

