# Supplementary material for: Vision protection and robust axon regeneration in glaucoma models by membrane-associated Trk receptors
Source: Mol Ther. 2022 Dec 5;31(3):810–24. doi: 10.1016/j.ymthe.2022.11.018 (PMC10014229; doi:10.1016/j.ymthe.2022.11.018)
Supplement: Document S1. Figures S1 and S2 and Table S1 [file mmc1.pdf]

YMTHE, Volume *31*

Supplemental Information

Vision protection and robust axon regeneration in glaucoma models by membrane-associated Trk receptors

Euido Nishijima, Sari Honda, Yuta Kitamura, Kazuhiko Namekata, Atsuko Kimura, Xiaoli Guo, Yuriko Azuchi, Chikako Harada, Akira Murakami, Akira Matsuda, Tadashi Nakano, Luis F. Parada, and Takayuki Harada

Supplementary Information

Supplementary Figures S1, S2 and Figure legends

Supplementary Table S1

Figure S1

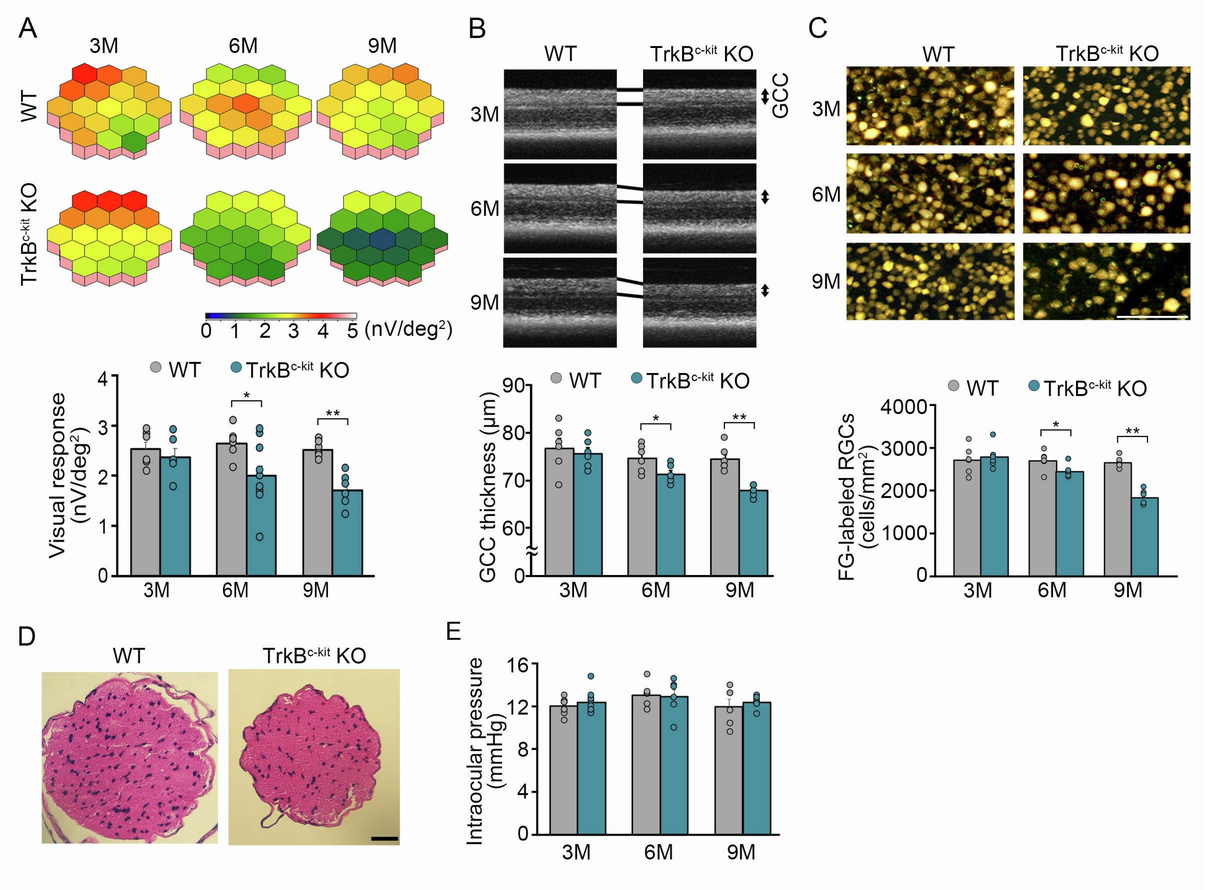

Figure S1. Neuron-specific TrkB deficiency leads to glaucomatous retinal and optic nerve degeneration in aged mice

(**A**) Multifocal electroretinography (mfERG) of WT and TrkB$^{c\text{-}kit}$ KO mice at 3, 6, and 9 months (M) of age. Retinal responses of second-order kernel are presented with 3D plots images (upper panel) and quantitative analyses of the retinal response amplitude are shown (lower panel). Retinal responses in TrkB$^{c\text{-}kit}$ KO mice were comparable to those in WT mice at 3 M, but they were significantly reduced by 6 and 9 M compared with WT mice. The one-way ANOVA with Tukey-Kramer post hoc test was used. n = 6-10 per group. *$p < 0.05$, **$p < 0.01$. (**B**) Optical coherence tomography (OCT) of WT and TrkB$^{c\text{-}kit}$ KO mouse retinas (upper panel), and evaluation of the thickness of the ganglion cell complex (GCC) at 3, 6, and 9 M of age (lower pane). Imaging with OCT revealed that GCC thickness in TrkB$^{c\text{-}kit}$ KO mice decreased

progressively with time, whereas there was no change in WT mice. The one-way ANOVA with Tukey-Kramer post hoc test was used. n = 6 per group. *$p < 0.05$, **$p < 0.01$.

(**C**) Retrograde labelling of retinal ganglion cells (RGCs) in WT and $TrkB^{c\text{-}kit}$ KO mice (upper panel), and quantitative analysis of Fluorogold (FG)-labelled RGCs (lower panel) at 3, 6, and 9 M of age. RGC number in $TrkB^{c\text{-}kit}$ KO mice was significantly decreased compared with WT mice at 6 and 9 M. The one-way ANOVA with Tukey-Kramer post hoc test was used. n = 6 per group. *$p < 0.05$, **$p < 0.01$. Scale bar, 100 µm.

(**D**) Hematoxylin and eosin staining of WT and $TrkB^{c\text{-}kit}$ KO mouse optic nerves. Thinning of the optic nerve was observed in $TrkB^{c\text{-}kit}$ KO mice at 9 M. Scale bar, 25 µm.

(**E**) Intraocular pressure of WT and $TrkB^{c\text{-}kit}$ KO mice. Both WT and $TrkB^{c\text{-}kit}$ KO mice at 3, 6, and 9 M showed no changes in intraocular pressure with aging. n = 6-10 per group.

Figure S2

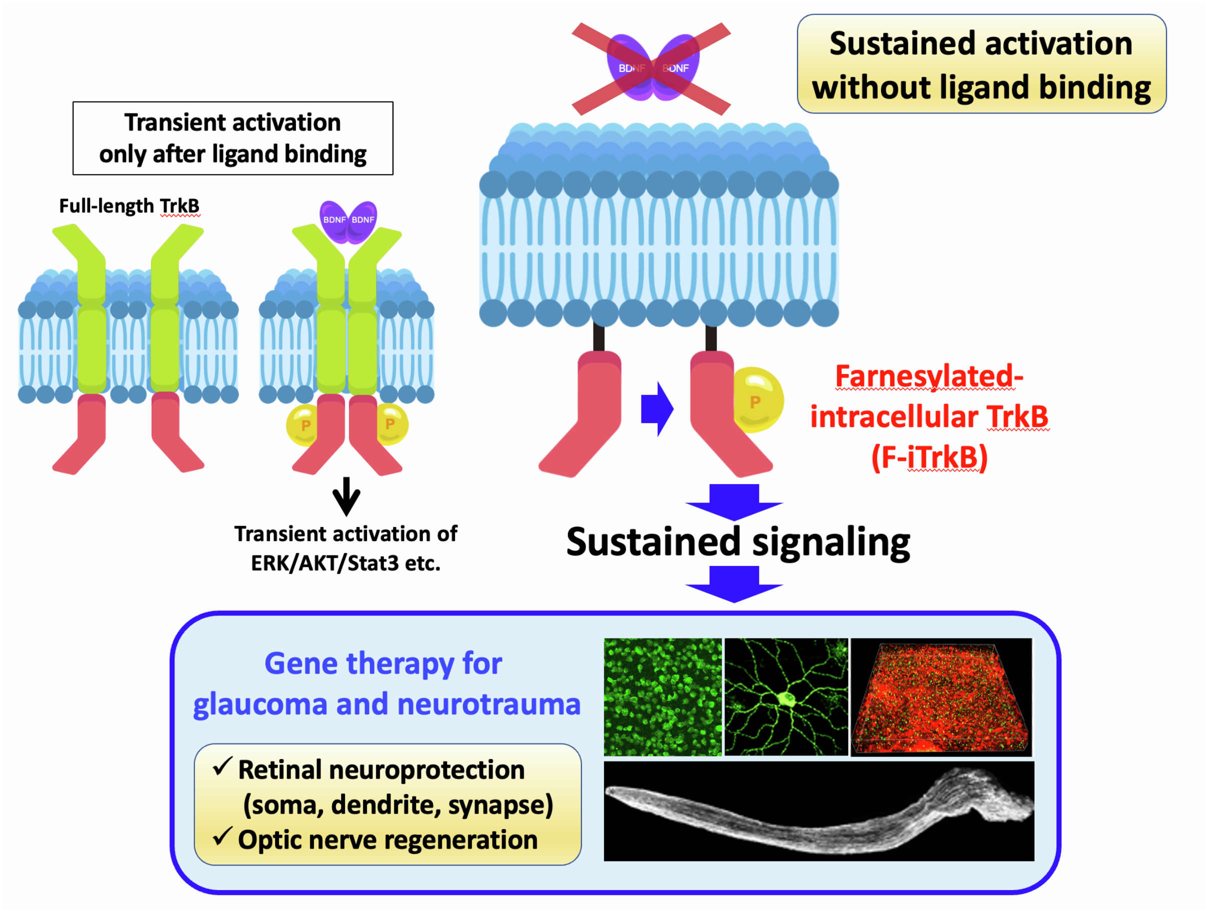

Figure S2. Schematic model of sustained activation of TrkB signaling without BDNF

AAV-mediated delivery of farnecylated-intracellular TrkB (F-iTrkB) induced sustained activation of the downstream signaling of full-length TrkB (ERK/AKT/Stat3 etc.) in retinal ganglion cells (RGCs) without BDNF. Intraocular injection of AAV-F-iTrkB induced RGC protection and robust optic nerve regeneration in mouse models of glaucoma and optic nerve injury.

Table S1. Reagents or resources used in the present study

Reagent or resource	Designation	Source or reference	Identifiers
Antibody	Alexa Fluor 488 Donkey anti-Guinea Pig	Jackson Immuno Research	AB_2340472
Antibody	Alexa Fluor 488 Donkey anti-Mouse	Thermo Fisher	A21202
Antibody	Alexa Fluor 488 Donkey anti-Rabbit	Thermo Fisher	A21206
Antibody	Alexa Fluor 568 Goat anti-Mouse	Thermo Fisher	A11004
Antibody	Alexa Fluor 568 Goat anti-Rabbit	Abcam	ab175471
Antibody	Alexa Fluor 647 Donkey anti-Rabbit	Abcam	ab150075
Antibody	Guinea Pig anti-RBPMS	MERCK	ABN1376
Antibody	anti-actin	BD Bioscience	612656
Antibody	anti-BNPI	Santa Cruz	#sc-377425
Antibody	anti-c-Myc	Santa Cruz	sc-40
Antibody	anti-ERK2	BD Bioscience	610103
Antibody	anti-GRB2	Transduction	G16720(610112)
Antibody	anti-HA	Biolegend	901533
Antibody	anti-Neurofilament H (clone SMI32)	Biolegend	#801702
Antibody	anti-Akt	Cell Signaling Technology	#9272
Antibody	anti-GSK-3β	MERCK	07-1413
Antibody	anti-p38	Cell Signaling Technology	#4511S
Antibody	anti-Phospho 44/42 MAPK (Erk1/2)	Cell Signaling Technology	#4695
Antibody	anti-Phospho Akt (Ser473)	Cell Signaling Technology	#9271
Antibody	anti-Phospho GSK-3β (Ser9)	Cell Signaling Technology	#9336
Antibody	anti-Phospho Stat3 (Ser727)	Bios Antibodies	bs-3429R
Antibody	anti-Phospho-p38 MAPK (T180/Y182)	Santa Cruz	sc-535
Antibody	anti-Phospho-Stat1 (Tyr701)	Cell Signaling Technology	#7649
Antibody	anti-Phospho-TrkB(Y515)	Bioworld Technology	BS4200
Antibody	anti-PSD95	Santa Cruz	#sc-377425
Antibody	anti-Stat1	Cell Signaling Technology	#9172
Antibody	anti-Stat3	Cell Signaling Technology	#12640
Chemicals	Glutathione sepharose4B resin	Thermo Fisher Scientific	17-0756-01
Recombinant protein	Alexa-Fluor 647 conjugated cholera toxin beta subunit	Thermo Fisher Scientific	C34778
Cell line	Human: HEK293T cells	TAKARA	632273
Cell line	Neuro2A cells	ATCC	CCL-131
Cell line	Cos-7 cells	ATCC	CRL-1651
Strain, strain background (Mus musculus)	Mice: PTEN flox/flox	Jackson	4597
Strain, strain background (Mus musculus)	Mice: TrkB flox/flox, c-kit-Cre+	Harada et al., 2011	N/A
Strain, strain background (Mus musculus)	Mice: Ribo-Tag	Jackson	11029
Recombinant DNA	piTrkB-WPRE-hGH	Visual Research Project	N/A
Recombinant DNA	pF-iTrkA-WPRE-hGH	Visual Research Project	N/A
Recombinant DNA	pF-iTrkB-WPRE-hGH	Visual Research Project	N/A
Recombinant DNA	pF-igp130-WPRE-hGH	Visual Research Project	N/A
Recombinant DNA	pF-iLIFR-WPRE-hGH	Visual Research Project	N/A
Recombinant DNA	pF-KD-iTrkB-WPRE-hGH	Visual Research Project	N/A
Recombinant DNA	pTM-iTrkB-WPRE-hGH	Visual Research Project	N/A

Recombinant DNA	pGST-F-KD-iTrkB-WPRE-hGH	Visual Research Project	N/A
Recombinant DNA	pRC2-mi342	TAKARA	6652
Recombinant DNA	pHelper	TAKARA	6652
Recombinant DNA	pAAV-CAG-F-iTrkB-WPRE-hGH	Visual Research Project	N/A
Recombinant DNA	pAAV-CAG-F-iTrkA-WPRE-hGH	Visual Research Project	N/A
Recombinant DNA	pAAV-CAG-GFP-WPRE-hGH	Visual Research Project	N/A
Recombinant DNA	pAAV-CB-F-GFP-WPRE-hGH	Visual Research Project	N/A
Recombinant DNA	pAAV-CAG-Cre	Visual Research Project	N/A
Software	Image J	NIH, Bethesda, Maryland	https://imagej.nih.gov/ij/
Software	Imaris	Bitplane	Imaris x64 Ver.9.2.1
Software	Adobe Photoshop CC	Adobe	https://www.adobe.com/products/photoshop.html
Software	Bowtie v2.2.3	Langmead & Salzberg, 2012	http://bowtie-bio.sourceforge.net/index.shtml
Software	TopHat v2.0.12	Kim et al., 2013	https://ccb.jhu.edu/software/tophat/index.shtml
Software	HTSeq v0.6.1	Anders et al., 2015	https://htseq.readthedocs.io/en/master/
Software	DESeq R package	Anders & Huber, 2010	http://bioconductor.org/packages/release/bioc/ html/DESeq.html
Software	KOBAS	Mao et al., 2005	http://kobas.cbi.pku.edu.cn/kobas3/downl/oad/
Software	JMP ver15.2.0	SAS Institute Inc	https://www.jmp.com/ja_jp/home.html
Other	FV3000	OLYMPUS	N/A
Other	Optomotory	Cerebral Mechanics Inc.	1070
Other	VERIS 6.0 system	Electro-Diagnostic Imaging, Inc.	https://www.veris-edi.com/
